# Natural Selection on Functional Modules, a Genome-Wide Analysis

**DOI:** 10.1371/journal.pcbi.1001093

**Published:** 2011-03-03

**Authors:** François Serra, Leonardo Arbiza, Joaquín Dopazo, Hernán Dopazo

**Affiliations:** 1 Evolutionary Genomics Lab, Bioinformatics & Genomics Department, Centro de Investigación Príncipe Felipe, Valencia, Spain; 2 Functional Genomics Lab, Bioinformatics & Genomics Department, Centro de Investigación Príncipe Felipe, Valencia, Spain; 3 CIBER de Enfermedades Raras (CIBERER), Centro de Investigación Príncipe Felipe, Valencia, Spain; 4 Functional Genomics Node (INB), Centro de Investigación Príncipe Felipe, Valencia, Spain; University of Zurich, Switzerland

## Abstract

Classically, the functional consequences of natural selection over genomes have been analyzed as the compound effects of individual genes. The current paradigm for large-scale analysis of adaptation is based on the observed significant deviations of rates of individual genes from neutral evolutionary expectation. This approach, which assumed independence among genes, has not been able to identify biological functions significantly enriched in positively selected genes in individual species. Alternatively, pooling related species has enhanced the search for signatures of selection. However, grouping signatures does not allow testing for adaptive differences between species. Here we introduce the Gene-Set Selection Analysis (GSSA), a new genome-wide approach to test for evidences of natural selection on functional modules. GSSA is able to detect lineage specific evolutionary rate changes in a notable number of functional modules. For example, in nine mammal and *Drosophilae* genomes GSSA identifies hundreds of functional modules with significant associations to high and low rates of evolution. Many of the detected functional modules with high evolutionary rates have been previously identified as biological functions under positive selection. Notably, GSSA identifies conserved functional modules with many positively selected genes, which questions whether they are exclusively selected for fitting genomes to environmental changes. Our results agree with previous studies suggesting that adaptation requires positive selection, but not every mutation under positive selection contributes to the adaptive dynamical process of the evolution of species.

## Author Summary

Characterizing genome adaptation is paramount for understanding evolutionary genomics. Classically, the search for positively selected genes has been used to identify adaptive differences in morphology, physiology and behavior between species. However, this approach assumed gene independence and was unable to identify sets of functions significantly enriched by positively selected genes. To overcome such limitation, we apply an alternative test on the evolutionary rates of genes, called Gene-Set Selection Analysis (GSSA), which is able to detect functional sets of genes evolving at high and low evolutionary rates in genomes. Our analysis illustrates that by focusing on sets of genes instead of individual loci, we are able to describe a richer relationship between positive selected genes and the adaptive evolution of functions in different genomes. For example, GSSA identified many positively selected genes within biological functions under strong evidence purifying selection in mammals and *Drosophilae*; or an almost equal distribution of positively selected genes in functions evolving at significantly high and low rates in primates. Such findings show the complex correspondence between positive selection and the dynamic process of adaptive evolution in genomes.

## Introduction

Adaptation analysis at a large or genome scale relies on methods and concepts originally conceived for the study of single genes (i.e.: positively selected genes, PSGs). The current paradigm for large scale analysis of adaptation typically involves conducting a given test individually for all of the genes of a genome in order to find those with statistically significant deviations from neutrality (that is, a significant increase above a threshold value of the nonsynonymous to synonymous rate ratio ω  =  dN/dS  =  1) [1]. Nominal *p*-values obtained in this way require the adjustment for multiple testing to derive the definitive list of PSGs. In a second step, a conventional functional enrichment test [2], [3] is applied to detect if functional modules are significantly enriched by PSGs. The test ascertains the overabundance of modules of functionally related genes (e.g. GO: gene ontology, KEGG: the Kyoto Encyclopedia of Genes and Genomes pathways, etc.) in the resulting list of PSGs. With variations in the methods chosen to test for positive selection and/or to search for functional enrichment, this threshold-based approach has been applied in different comparative genomic studies [4], [5], [6], [7] with results falling below the initial expectation. In fact, the few functional modules apparently under selection hardly ever reached statistical significance in single species after correcting for multiple testing.

To circumvent this statistical problem recent works have drawn their conclusions by looking for signatures of selection in related groups of species [8], [9], [10]. Specifically, by modeling heterogeneous rates across sites, functional modules with significantly elevated ω values (not necessarily containing PSGs) were described in 12 *Drosophila* genomes [8]. Categories showing significant deviations included *defense response, proteolysis, DNA metabolic process,* and *odorant binding,* among others. In the analysis of 6 mammalian genomes [9], *chemosensory perception* and *defense/immunity related processes* were functionally enriched after pooling together all PSGs (400 genes) in primates and rodents respectively. Finally, using the deviations from the expected branch length on gene trees, similar patterns of selection across genomes were found for a group of gamma proteobacteria [10]. Although the strategy of pooling signatures across species has shown sufficient statistical power to describe adaptive functional differences, it fails to offer a solution for testing adaptive functional events occurring in independent lineages after speciation [8], [9], [10].

The limitations of methods based on *a prior* threshold application have already been noticed in other omics fields such as transcriptomics [11], and have successfully been overcome by gene-set based methods [2], [12]. These kinds of methods, regularly applied in the field of functional genomics [2], [12] can be used to search for quantitative differences in evolutionary rates among functional modules of individual genomes. The hypothesis we aim to test here is not about individual genes, but about functional modules. Mutations occur at DNA level but selection acts on phenotypes modifying gene frequencies that finally accounts for functional properties of cells [13]. Most mutations in genes either remain finally fixed or disappear because of their beneficial or disadvantageous effect, respectively. This effect on the function of individual proteins can only be understood in the context of the system in which proteins are involved (e.g. a pathway, GO functional roles, etc.). If a list of genes arranged by some parameter that accounts for their evolutionary rates is examined, it is expected that genes belonging to pathways or functional classes favored or disfavored by selection will tend to appear towards the extremes.

Here we set forth the Gene-Set Selection Analysis (GSSA), a gene-set based test that searches for significant evidences of the action of natural selection modeling the evolutionary rates of groups of genes in genomes. Two different and widely accepted definitions of functional modules: GO [14] terms and KEGG [15] pathways have been used on the genomic coding sequences of five mammals and six *Drosophila* species. By using this gene-set strategy we found a large number of functional modules that have significantly increased or decreased their rates of evolution with respect to the ancestral state. We will show evidences of selection working in groups of functionally related genes, suggesting that they share a common pattern of evolution imprinted by natural selection. In addition, all biological GO processes previously found as significantly enriched by PSGs were distinguished within the set of functions evolving at higher rates than the expected in genomes. Finally, the relationship between GSSA results and the relative influence of PSGs during adaptive evolution is discussed.

## Results

### Gene-set selection analysis on functional modules

**Figure 1:**
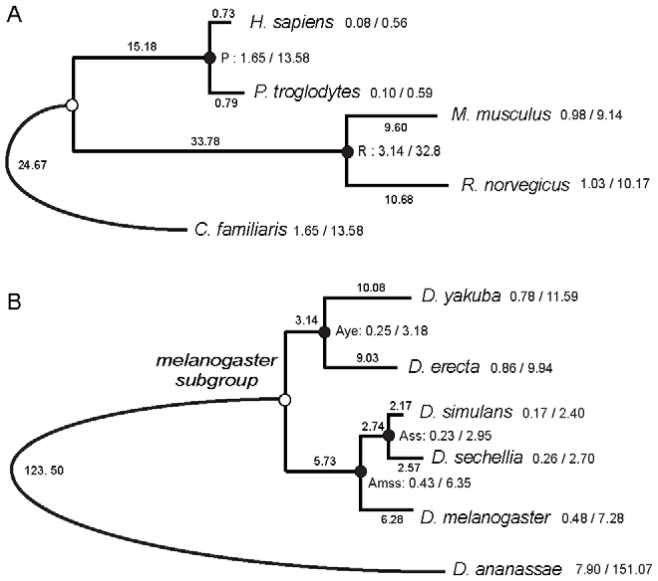
Mammal and Drosophila phylogenies. Numbers on internal and external nodes represent the median number of nonsynonymous and synonymous substitutions per codon (dN/dS) estimated from all the coding sequences compared in mammal (A) and Drosophila (B) genomes. Branch lengths and rates were multiplied by 100. Ancestral estimation of parameters was done in primates (P), rodents (R), *D. yakuba* and *D. erecta* (Aye), *D. simulans* and *D. sechellia* (Ass), and *D. melanogaster, D. simulans* and *D. sechellia* (Amss). *C. familiaris* and *D. ananassae* were chosen as outgroup species in the corresponding tree.

**Figure 2:**
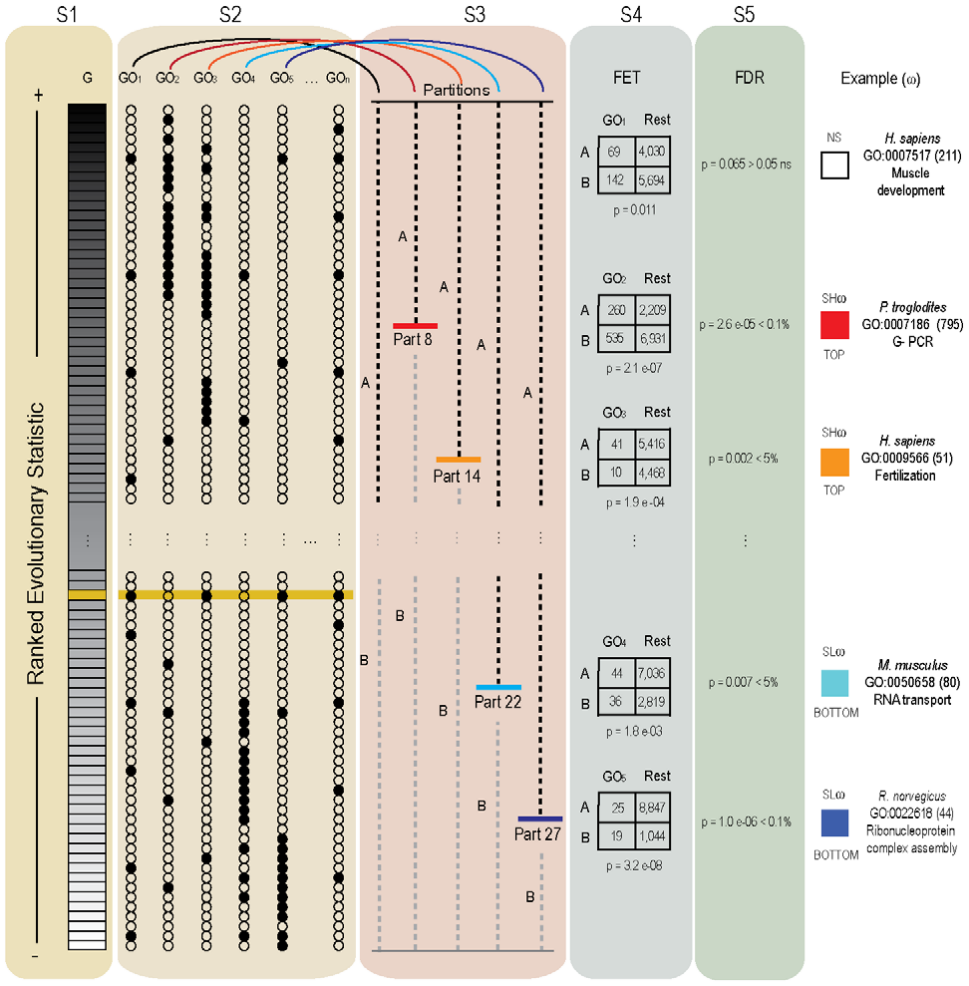
Summary of the steps developed by the GSSA. GSSA can be roughly described in a series of five steps (S1 to S5). S1: rank genes of a genome according to an evolutionary variable, S2: assign functional classes to all the listed genes, S3: apply a fixed number of partitions on the ranked list, S4: proceeds with a Fisher exact test (FET) for each partition, S5: adjust p-values by FDR. See text for a full description. Colored boxes (red, orange, cyan and blue) represent functional modules with genes significantly accumulated (0.1% FDR and 5% FDR) at the corresponding extremes of a list (top and bottom), and therefore with significantly high (SH) and low (SL) values of the evolutionary variable (ω) respectively. White represents a non-significant association (NS). Examples show five alternative GO categories with significant and non-significant distributions of the ω statistic. In parenthesis, the total number of genes corresponding to the GO term is shown. For GO1, the function seems to be uncorrelated with the arrangements of the genes. In the example (GO:0007517) partition 16 in human (not shown in the picture) reported the lowest *p*-value (p = 0.011) although it was not significant after FDR correction (FDR = 0.065). Upper (A) and lower (B) sides of the ranked list (S3) represent both sides of the specified partition number. Remainder GO categories (GO2 to GO5) show the association of dark dots with values located at the top (significant high ω values –SHω), and at the bottom (significant low ω values –SLω) of the list (for GO2-GO3 and GO4-GO5, respectively). In examples, FETs found the most significant p-value for partitions 8, 14, 22 and 27 for GO:0007517, GO:0007186, GO:0009566, GO:0050658 and GO:0022618 in chimpanzee, human, mouse and rat genome, respectively.

Mammals, represented by human, chimpanzee, rat and mouse, and five Drosophila genomes were studied. For each species, genes were ranked into four lists according to the estimation of i- synonymous (dS), ii- nonsynonymous (dN) rates of substitution, iii- selective pressures (ω = dN/dS), and iv- the change of selective pressures between (A) ancestor and (D) descendant species (Δω_D_ = ω_D_−ω_A_) along the phylogeny (Figure 1). Maximum likelihood (ML) estimates of evolutionary variables were performed using a free-ratio branch model [16]. As such, four lists containing 12,543 and 9,240 orthologous genes in mammals in *Drosophila* species were obtained for the analyses, respectively. GSSA was conducted using a total of 1,394/199 and 1,331/116 GO/KEGG terms in mammals and *Drosophila* species respectively. GSSA is performed in five different steps (S1 to S5 in Figure 2). First, the method ranks all genes within a genome (G) according to one of the alternative evolutionary variables (dS, dN, ω and Δω). Second, genes are associated (dark dots) to different functional categories (GO or any other functional term). Note that a single gene can be associated with multiple functions (yellow bar in Figure 2). Third, for each functional category a total of 30 partitions are established along the list of ranked values [17], [18]. Fourth, for each partition GSSA computes a two-tailed Fisher's exact test and reports significant over or under represented functional classes comparing the upper side (A) and the lower side (B) of the list. Finally, *p*-values are corrected for multiple testing (FDR). Throughout the manuscript only *p*-values for partitions with the highest confidence were reported after FDR.

The application of GSSA to lists of genes ranked by dS, dN, ω and the Δω values yielded a large number of functional modules (defined by GO and KEGG annotations) with rates that were significantly skewed toward the extremes of the lists (Table 1) in mammal and *Drosophila* species. For instance, 11% of GO terms, and 15% of KEGG pathways contain genes with biased distribution of rates towards the top of the ranked list, and found statistically significant at high ω ratio (SHω, 5% false-discovery rate, FDR) in mammals. Alternatively, 4.1% and 2.6% of GO terms and KEGG pathways were found with significantly high values of ω (SHω) in *Drosophila*, respectively.

**Table 1:** Numbers and percentages of functional modules with significant results after GSSA.

		SH*		SL*	
		KEGG	GO	KEGG	GO
Mammals	dS	15 (1.9)	187 (3.3)	12 (2.1)	364 (6.5)
	dN	145 (18.2)	708 (12.6)	230 (28.9)	1,839 (32.9)
	ω	123 (15.5)	649 (11.6)	206 (25.9)	1,675 (30.0)
	Δω	64 (8.0)	421 (7.5)	107 (13.4)	818 (14.7)
Drosophilas	dS	18 (3.1)	104 (1.5)	26 (4.5)	1,263 (18.9)
	dN	31 (5.3)	276 (4.1)	26 (4.5)	2,097 (31.5)
	ω	15 (2.6)	213 (4.1)	24 (4.1)	1,321 (19.8)
	Δω	2 (0.3)	143 (2.1)	7 (1.2)	184 (2.8)

GO/KEGG terms were 1,394/199 in mammals and 1,331/116 in Drosophilas.

* Statistically significant high (SH) and low (SL) rates after the GSSA (5% FDR).

Table 1 also reveals that functional modules with genes changing at significantly low ω ratios (SLω), and therefore showing a distribution shifted towards the bottom of the ranked list (see Figure 2), were more frequent than modules under the significantly high ω (SHω). This observation is in agreement with the fact that purifying selection is the predominant form of selection in biological systems. Moreover, in support of the slightly neutral character of synonymous mutations, and the effects of population size in the final outcome of selection [19] GSSA results show a higher number of significant deviations of dS in *Drosophila* rather than in mammals.

Only a minor proportion of functional terms changed significantly at higher or lower rates relative to estimates of the corresponding ancestral lineages. Specifically, increased or decreased ω values on the external branches (recorded by positive and negative values of Δω) were observed for only half of the cases where a significant increase or decrease of ω was identified in mammals and *Drosophilas*. This observation points out the conservative character of the selective constraints in functional related groups of genes during evolution.

**Figure 3:**
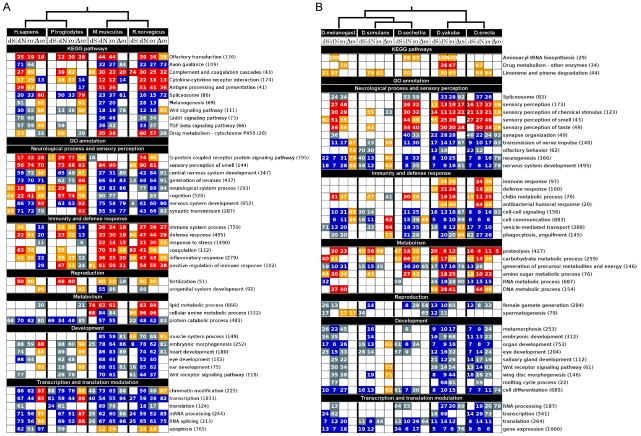
GSSA of evolutionary variables. The figure shows a selection of GO terms and KEGG pathways with significant and not significant deviations after GSSA of evolutionary rates in mammals (A) and Drosophila (B) species. Colored boxes represent functional modules with genes significantly accumulated at the corresponding extremes of the ranked list as explained in Figure 2. The number inside each box represents the percentage of the total number of genes of the functional module (in parenthesis) that contribute to its significance. Here we reported the numbers of the first significant partition after FET and FDR. Topologies represent the phylogenetic relationships of species.

A summary of the results of the GSSA for mammals and *Drosophilas* is shown in Figure 3 (see Figures S1 to S4 for a complete description of results after GSSA in mammals and Drosophila species). The figure shows that GSSA has the power to detect many functional changes in evolutionary rates within a substantial number of functional categories. Although the rough pattern shows similar evolutionary constraints in groups of genes between the two main clusters of species, important differences were also detected within them. For instance, functional terms associated to *neurological process* and *sensory perception* clearly contrasted between primates and rodents (Figure 3A). While most of these terms are associated to a significant relative increase in rates from the common ancestor of primates (+Δω), all the changes observed in rodents were due to the relative increase of the selective constraints (-Δω) probably due to the effects of purifying selection from the common ancestor. Alternatively, functional modules associated to *Immunity* and *Defense response* evolved at significantly higher rates than expected in rodents, but decreased significantly in relation to the ancestral rates in primates. Such functional differences between primates and rodents were previously observed when pooling groups of species [9]. Other functional modules such as *Development*, and *Transcription/Transduction* comparatively evolved at very low dN and ω ratio but experienced a higher relaxation of the ancestral constraints (+Δω) in primates than in rodents. Moreover, significant differences in rates can be detected between human and chimpanzee (Ha04360: *Axon guidance*, Ha04610: *Antigen processes and presentation,* GO0007268: *synaptic transmission*, among others), and between mouse and rat (GO0007186: *G-protein coupled receptor protein signaling pathway*, and Ha04310: *Wnt signaling pathway*, among others).

In addition, most of the GO terms significantly associated to high dN and ω in *Drosophilas* were unevenly distributed within the two clusters of the phylogeny (Figure 3B). GO terms such as *sensory perception, defense response, immune response* and *metabolic process,* among others, presented a remarkable divergence in the monophyletic groups of *D. erecta* and *D. yakuba* but they were not observed in *D. sechellia, D. melanogaster* and *D. simulans*. Most of GO terms from *Development, Transcription* and *Translation* (Figure 3A and 3B) were significantly accumulated towards the extremes of the lists corresponding to the lowest rates of substitutions, suggesting they are significantly constrained by strong purifying selection (5% FDR) in both taxa.

The fact that most of the functional modules under selection (SHω and SLω) correlate with changes in dN, suggests that selective pressures are mainly driven by nonsynonymous rather than by synonymous substitutions during evolution. Moreover, according to the expectation of the nearly neutral theory, a low but still considerable number of significant associations of functional modules to dS were found in *Drosophila* (19.5%) and rodents (11.3%), while in primates (6.4%), where population sizes are known to be smaller, the number of significant modules was smaller [20].

The strategy presented here lead to detect significant patterns of increments and decrements modeled by natural selection in evolutionary rates of functional groups of genes. This pattern is consistent with the hypothesis that natural selection acts on phenotypes by the combined action of many functional related genes. Moreover, this functionally based approach identified with statistical significance, and on individual species, all the functional modules previously found significantly enriched by positively selected genes and therefore the main targets of adaptive biological functions in species (Table 2) (see Supplementary Table S3 for a complete list of terms). Although GSSA is not a test for positive selection, it is evident that functional modules containing PSGs can be significantly detected by this method on individual species. In the next section we will analyze the relative contribution of PSGs to the statistical differentiation of functional modules in genomes.

**Table 2:** Functional enrichment results using gene-by-gene and gene-set approaches.

Biological process	Functional category enriched by PSGs (Reference #)							GSSA results	
	1	2	3	4	5	6	7	SHω	SLω
Olfaction/Sensory perception of smell	H	Pr**				Pr**		H**, C**, M**, R**, Dmel*, Dsec*, Dere**, Dyak**	
Chemosensory perception	H	Pr**						H**, C**, M**, R**, Dmel**, Dsec**, Dere**, Dyak**	
G-protein-mediated signaling	H				H	Pr**		H**, C**, R*	
Proteolysis							Ds	M**, R**, Dmel**, Dsim*, Dsec*, Dyak**, Dere**	
Immune response		Pr**		H, C		Ro**		C*, M**, R**, Dyak*, Dere*	
Inflammatory response						Ro**		H*, C*, M**, R**	
Defense response						Ro**		H*, C*, M**, R**, Dyak**, Dere*	
Response to wounding						Ro**		H*, M**, R**	
T-cell-mediated immunity		Pr**						M*	
Natural killer-cell-mediated immunity		Pr*						R*	
B-cell- and antibody-mediated immunity		Pr*						M**, R**	
Response to pest, pathogen, or parasite				H				C*, M**, R**, Dyak*, Dere*	
Stress response					C	Ro**		M**, R**	
Cell surface receptor-mediated signal transduction	H					Pr**		C*	Dmel*, Dyak*, Dere*
Cell adhesion	H							R*	H**, C**, Dmel**, Dere*
Signal transduction/intracellular signaling cascade	H, C		Pr				Ds		H**, C**, M**, R**, Dmel**, Dsec*, Dyak**, Dere**
Ion transport	H				H		Ds		H*, M**, R**, Dmel*, Dsec*, Dere*
Potassium ion transport			Pr						H*, C*, M**, R**
Protein transport				H			Ds		H*, C**, M**, R**, Dmel**, Dsim*, Dsec**, Dere**, Dyak**
Protein metabolism & modification				H, C	C		Ds		H**, C**, M**, R**, Dere*, Dyak*
Nervous system development							Ds		H*, M**, R**, Dmel**, Dsec*, Dyak**, Dere**
Organ development							Ds		H*, M**, R**, Dmel**, Dsec*, Dyak**, Dere**
Post-embryonic development							Ds		M*, Dmel*, Dyak**, Dere*
Cell proliferation and differentiation	C						Ds		H**, C*, M**, R**, Dmel**, Dsec*, Dyak**, Dere**
Inhibition of apoptosis		Pr*							H*, Dyak*
Transcription				H, C	C		Ds		H**, C**, M**, R**, Dere*

The table depicts some selected biological functions enriched by PSGs as cited in references 1 to 7, and the corresponding significant result observed after GSSA of ω values. References 1 to 7 correspond to cites 6, 7, CSAC, 4, 5, 9 and 8 in the manuscript, respectively. Abbreviations: SHω: statistically significant high ω values; SLω: statistically significant low ω values; H: *H. sapiens*; C: *P. troglodytes*; Pr: primates; M: *M. musculus*; R: *R. norvegicus*; Ro: rodents; Dmel: *D. melanogaster*; Dsim: *D. simulans*; Dsec: *D sechelia*; Dyak: *D. yakuba*; Dere: *D. erecta;* Ds: *Drosophila species.*

*: *p<0.05;*

** *p<0.001. CSAC: Chimpanzee Sequencing and Analysis Consortium, Nature. 2005 vol. 437 (7055) pp. 69*–*87.*

### Positively selected genes and the evolution of functional modules

**Figure 4:**
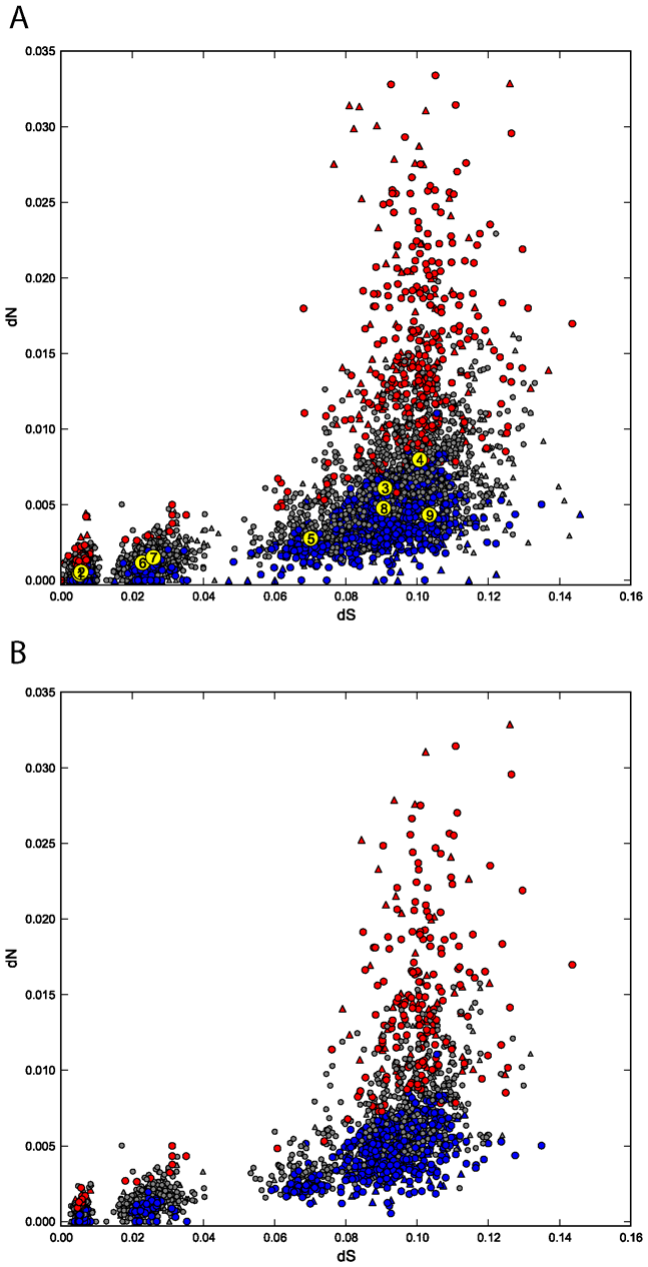
Positive selection and evolution of functional modules. Circles and triangles represent the median values of dN and dS for KEGG pathways and GO terms (level 6–7), respectively in mammals, and in the *Drosophila* species. Functional modules with SHω and SLω results after GSSA are shown in red and blue. Those modules without statistical differences are gray. Yellow dots depict the median dS and dN values for *H. sapiens* (1), *P. troglodytes* (2), *M. musculus* (3), *R. norvegicus* (4), *D. simulans* (5), *D. sechellia* (6), *D. melanogaster* (7), *D. yakuba* (8) and *D. erecta* (9). (B) In this case, circles and triangles represent a subset (of A) with modules containing at least one PSG. Note that they are distributed along a wide range of values of dS and dN and in functional categories with significant (red/blue), and non-significant (gray) results after the GSSA (ω ratio).

GSSA tests for differences in rates over functional related groups of genes. To what extent genes under positive selection contribute to the significance of functional modules in mammals and Drosophila species after GSSA? To answer this question, branch-site (the most sensitive) test of positive selection was conducted on terminal branches of phylogenies (Figure 1). We found 715 PSGs in mammals and 626 in *Drosophila*. Figure 4A shows the distribution of the mean evolutionary rates (dN and dS) of functional modules providing significant and not significant results after GSSA of the w ratio. When considering the total number of the functional modules with PSGs, 55%, 53%, and 42% of these original functional categories observed with SH, SL and NS results after GSSA (ω values) still remained (Figure 4B). This suggests that: 1- evolution of many of the functional modules changing at SHω ratios in the genome is not driven by a considerable accumulation of PSGs. Functional modules such as *complement and coagulation cascades* in human, *gonad development* in chimpanzee, *regulation of innate immune response* in mouse, *primary immunodeficiency* in rat, and *spermatid differentiation* in *D. melanogaster* are examples of functional modules evolving at significantly elevated ω ratio without any PSGs; 2- molecular adaptation takes place in functional modules under strong selective constraints (see last part of Table 2). For instance, *apoptosis* in human, *generation of neurons* in chimpanzee, *tissue development* in mouse, *Wnt signaling pathway* in rat, *eye development* in *D. melanogaster*, *wing disc development* in *D. yakuba,* and *generation of neurons* in *D. erecta* are some of the functional modules evolving at SLω ratios in the corresponding genomes that contain PSGs; and finally, 3- an important number of functional modules without significant differences in ω ratios (grey dots in Figure 4) still contain genes under positive selection. For instance, *homologous recombination* in humans, *brain development* in chimpanzee, *female* or *male sex differentiation* in mouse, *regulation of mitotic cell cycle* in rat, *chromatin modification* in *D. sechellia,* and *oogenesis* in *D. melanogaster.* These results are in agreement with previous observations in *Drosophila* were it was emphasized that not every mutation under positive selection responds to a change in selection [21]. Beneficial changes could occur at evolutionary equilibrium, repairing previous deleterious changes and restoring existing functions [21].

Finally, we ask if PSGs preferentially concentrate in functional modules evolving at faster rates in different genomes. For doing that we computed the mean number of PSGs in functional modules with SHω and SLω results (red and blue dots in Figure 4B). As expected, functional modules evolving at high ω ratio contain higher numbers of PSGs in rodents (*p*≪0.01), mammals (*p*≪0.01), and *Drosophila* (*p*≪0.01) species. For primates however, it was not significant (*p = *0.47), indicating that PSGs are distributed almost evenly in functional modules evolving at significantly high and low values of ω in human and chimpanzee.

To contrast these results, PSGs from previous works in mammal and Drosophila species were collected [8], [9]. The pattern of distribution of PSGs in functional modules was in agreement with the mentioned results: significantly skewed (*p*≪0.01) towards higher numbers of PSGs in mammals, rodents, and *Drosophila* species, but showing no differences in primates (*p = *0.73).

In summary, PSGs are frequently observed in functional modules evolving under a wide range of evolutionary scenarios; however, they concentrate more frequently in functional groups of genes changing at elevated rates in rodents and *Drosophila* species. Alternatively, PSGs were evenly distributed in functional modules changing at the extreme rates of evolution in primates. This observation suggests that a more complex scheme than the cumulative differences of PSGs must rely on the observed adaptive differences in human and chimpanzee genomes. The search for integrative factors taking into account the action of multiple genes other than only those which have been targeted by positive selection [22], could provide a more accurate view for the analysis of the integrated framework underlying adaptation in complete genomes.

## Discussion

Evolutionary biologists recognize that natural selection works on phenotypes indirectly by changing the frequency of genes in populations [23]. Since the revolution of molecular techniques and its use in evolutionary genetics, the statistical search for adaptation at a gene level has superseded the complexity of measuring fitness in nature [24]. Nowadays, we look for adaptive evidences on genes and afterwards we search for over-represented functional modules among the list of PSGs found in the genomes. Given that tests which are generally employed assume independence in both steps, the cooperative action of the network of genes underlying phenotypes [22] is systematically disregarded [25]. The aim of the GSSA is not to test for evolutionary constraints on individual genes as has been addressed in several previous studies. GSSA tests for significant differences in rates over functionally related groups of genes and therefore, the relative contribution of a gene is weighed among all genes of the same functional module and their values compared with the general constraints observed in a genome. Many functional modules changing at elevated ω ratios will correspond to those previously described as functions significantly enriched by PSGs [6], [9] simply because many of the genes within that functional module were among those contributing towards statistical significance. In correspondence with the hypothesis that phenotypes change during evolution by the coordinated action of genes we provided evidences that natural selection changes evolutionary rates of many functional related genes in genomes. By using this strategy we increase the statistical power to search for biological functions that significantly change in rates during evolution.

The existence of many PSGs in functional modules evolving at significant low (or no-significant) ω ratios does not represent false positive results in the analysis of molecular adaptation. This observation, registered in our data and detected in previous publications, suggests that PSGs are frequently recruited in the genomes for other purposes than the classical increase of rates of functional set of genes compromised in adaptive processes such as evolutionary arm-races. A possible explanation is that many of the PSGs in the genomes are changing in association with the constraints imposed by the architecture of the network [26], or adjusting deleterious mutations of other genes of the network, just for the maintenance of its phenotypic function. In this sense, adaptation will requires positive selection, but not every mutation under positive selection contributes to the adaptive dynamical process of evolution of species [21].

Currently, with the possibility of conducting analysis at the genome level, evolutionary biology cannot disregard major aspects of systems biology approaches that consider the modular organization of genomes. With the testing strategy used here, we increased the statistical power for the evolutionary analysis on individual genomes and suggest that PSGs could have additional roles in the genome than the adaptive evolutionary change of phenotypes.

## Materials and Methods

### Orthologs selection, alignments, and filters

The subset of 23,438 known Ensembl human protein-coding genes of the Ensembl vs56.37a *H. sapiens* was retrieved from the Ensembl-Compara database vs56 [27]. All the human ortholog transcripts were retrieved for chimpanzee vs56.21l, mouse vs56.37i, rat vs56.34x, and dog vs56.2m. The subset of 14,076 known Ensembl *D. melanogaster* protein-coding genes of *D. melanogaster* was retrieved from the Ensembl Metazoa-Compara database vs4 [27]. Orthologs transcripts were retrieved from versions 56.13a of *D. simulans*, *D. sechellia*, *D. yakuba, D. erecta,* and *D. ananassae.*

DNA coding sequences (CDS) were aligned using the Muscle vs3.7 [28]. In mammals, the upper limit for dN and dS considered was those of the human interferon γ (dN = 3.06) and the relaxin protein [29] (dS = 6.39 substitutions per site per 1e9 years). Assuming the human–mouse, mouse-rat and human–chimp differentiation times to be about 80, 70 and 5 million years [30], respectively, ortholog comparisons between primates and rodents with dS≥1 and dN≥0.5, rodents with dS≥0.256, dN≥0.122, and primates with dS≥0.064 and dN≥0.030 substitutions/site were excluded. To improve alignments we run TrimAl [31] with heuristic method (-automated1) in Drosophila. Alignments smaller than 100 bp were excluded. The total number of alignments analyzed was of 12,453 and 9,240 in mammals and *Drosophila* respectively.

### Evolutionary analysis

Maximum likelihood estimation of dN, dS, and ω was computed using CodeML program from PAML[16]. Evolutionary rates were computed in orthologous sequences according to the free-ratio branch model assuming independent ω ratio for each branch of the tree of mammals and Drosophila species (see raw values of rates in Table S1 and S2). Evolutionary rates (dN, dS), its ratio (ω), and its difference between ancestral and descendant species (Δω) were ranked along all genes of genomes and further analyzed by GSSA.

External branches of Figure 1 were labeled as foreground to test for positive selection using branch-site models in Test I and Test II [32]. Positive results of relaxation of selective constraints (or weak signals of positive selection) were discarded [4]. To quantify the relative contribution of PSGs in functional modules showing SHω and SLω results in GSSA, a t-test (from R package [33]) with the mean number of PSGs per functional modules was computed in primates, rodents, mammals and Drosophila species. An independent set of PSGs was collected to test the robustness of our results in mammals [9], and *Drosophila* species [8].

### GSSA, evolutionary and statistical simulations

Gene-set selection analysis across lists of genes ranked by different evolutionary rate parameters (dS, dN, ω and Δω) was computed using the program Babelomics [34]. This program implements a version of GSA [17] which can be applied to any list of ranked genes regardless of the initial experimental design [2], [12]. The aim of the test is to find functional classes, namely blocks of genes that share some functional property, showing a significant asymmetric distribution towards the extremes of a list of ranked genes. This is achieved by means of a segmentation test, which consists on the sequential application of a Fisher's exact test over the contingency tables formed with the two sides of different partitions (A and B in Figure 2) made on an ordered list of genes. The two-tailed Fisher's exact test finds significantly over or under represented functional classes when comparing the upper side to the lower side of the list, as defined by any partition (in Figure 2, four of the five partitions show significant differences). Similarly to other equivalent gene-set analyses, the outcomes are those modules (GO and KEGG) significantly associated to high or low values of the evolutionary parameter used to rank the genes. Previous results showed that a number between 20 and 50 partitions often gives optimal results in terms of sensitivity and results recovered [18]. Here we applied 30 partitions along all the GSSA performed. Given that multiple functional classes (C) are tested in multiple partitions (P), the unadjusted p-values for a total of C×P tests were corrected by the widely accepted FDR method [35].

Originally, 1,394/1,331 GO terms, and 199/116 KEGG pathways were analyzed in mammals and *Drosophila* species respectively. The global GO directed acyclic graph was processed with Blast2GO [36] to extend the annotation at missing parental nodes, discarding GO levels out of 2 to 8 for mammals, and 2 to 12 for *Drosophilas*. The final set of GO and KEGG terms used in the GSSA corresponds to those containing a minimum number of 15 genes. To test possible biases attributed to the size of the functional category, the magnitude of change in evolutionary rate or the proportion of genes experiencing a rate change we randomized the original assignation of ENSG's to the list of ranked values and functional annotation (see Figure S5A). For each evolutionary variable and species 10.000 randomizations and the corresponding GSSA were performed. The proportion of false positives (significant results after GSSA) was computed for each evolutionary variable and plotted along the size of functional categories (from 20 to 1,400 with intervals of 20). Because this proportion never reached values higher than 0.5% (FDR) we rejected the possibility that either group size or rate distribution biased GSSA results in our data set (see Figure S5A and S5B-C).

Finally, in order to validate the independence of the GSSA from the effects of alternative evolutionary constraints we simulated selective regimes (purifying selection, positive selection and relaxation of selective constraints) using branch-site models. Here we addressed the possibility of a variation in the representation of significant results after GSSA (see Supplementary Figure S6). We found that when a massive enrichment of genes under each of the evolutionary scenarios described take place in the genome, none of them bias the results of GSSA (see Text S1).

## Supporting Information

Figure S1 Complete list of significant results of GSSA for GO terms in mammals. The figures cover from the most general to the most specific biological GO functions. GSSA (5% FDR) results for dS, dN, dN/dS & Δω using 731 GO terms.
(6.47 MB PDF)

Figure S2 Complete list of significant results of GSSA for GO terms in Drosophila species. The figures cover from the most general to the most specific biological GO functions. GSSA (5% FDR) results for dS, dN, ω & Δω using 386 GO terms.
(8.85 MB PDF)

Figure S3 Complete list of significant results of GSSA for KEGG pathways in mammals species. GSSA (5% FDR) results (82 KEGG pathways) for dS, dN, ω & Δω in mammals species.
(1.54 MB PDF)

Figure S4 Complete list of significant results of GSSA for KEGG pathways in Drosophila species. GSSA (5% FDR) results (43 KEGG pathways) for dS, dN, ω & Δω in Drosophila species.
(0.87 MB PDF)

Figure S5 Randomisation experiment. (A) The pipeline shows the steps followed to tests possible biases attributed to the size of the functional category, the magnitude of change in evolutionary rate and the proportion of genes experiencing a rate change in the GSSA. The proportion of false positive results never reached 5% (FDR) in mammals (B) and Drosophila (C).
(1.34 MB PDF)

Figure S6 Evolutionary and statistical simulation of GSSA. The pipeline shows the steps taken along three different spaces of analysis, the real data, the simulated data and the testing block. See Supplementary Results for a complete explanation of methods and results.
(1.42 MB PDF)

Table S1 Evolutionary rates of genes computed in Mammals. Complete values of evolutionary rates (dS, dN, ω & Δω) for all genes analysed.
(5.32 MB ZIP)

Table S2 Evolutionary rates of genes computed in Drosophila. Complete values of evolutionary rates (dS, dN, ω & Δω) for all genes analysed.
(9.55 MB XLS)

Table S3 The complete functional enrichment results using gene-by-gene and gene-set approaches. The table depicts all the biological functions enriched by PSGs as cited in references 1 to 7, and the corresponding significant result observed after GSSA of ω values. References 1 to 7 correspond to cites 6, 7, CSAC, 4, 5, 9 and 8 in the manuscript, respectively. Abbreviations: SHω: statistically significant high ω values; SLω: statistically significant low ω values; H: human; C: chimpanzee; Pr: primates; M: mouse; R: rat; Ro: rodents; Dmel: D. melanogaster; Dsim: D. simulans; Dsec: D sechelia; Dyak: D. yakuba; Dere: D. erecta; Ds: Drosophila species. *: p<0.05; ** p<0.001. CSAC: Chimpanzee Sequencing and Analysis Consortium, Nature. 2005 vol. 437 (7055) pp. 69–87.
(0.09 MB PDF)

Text S1 Supplementary Text S1.
(0.11 MB PDF)
