# Supplementary material for: Natural Selection on Functional Modules, a Genome-Wide Analysis
Source: PLoS Comput Biol. 2011 Mar 3;7(3):e1001093. doi: 10.1371/journal.pcbi.1001093 (PMC3048381; doi:10.1371/journal.pcbi.1001093)
Supplement: Figure S2 — Complete list of significant results of GSSA for GO terms in Drosophila species. The figures cover from the most general to the most specific biological GO functions. GSSA (5% FDR) results for dS, dN, ω & Δω using 386 GO terms. (8.85 MB PDF) [file pcbi.1001093.s002.pdf]

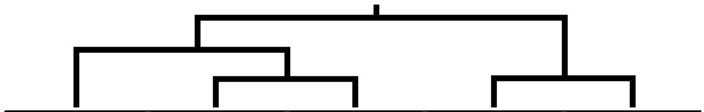

D. melanogast.				D. simulans				D. sechellia				D. yakuba				D. erecta				
dS	dN	ω	Δω	dS	dN	ω	Δω	dS	dN	ω	Δω	dS	dN	ω	Δω	dS	dN	ω	Δω	
37	10	22							20	22		17	18	26		12	13	16		GO:0030029 (122) actin filament-based process
8	6	18	80	15	12		59	60	6	33		7	8	15	92	6	7	6	92	GO:0048856 (1134) anatomical structure development
30	12	20							27	15	67	10	8	17	74	25	12	12	68	GO:0010926 (525) anatomical structure formation
40	31		62		13				9	36		10	14	9		8	10	17		GO:0007610 (257) behavior
	35	42											72				59	55		GO:0022610 (127) biological adhesion
11	5	13		15	11		59	28	6	24		5	7	10	88	5	5	5	75	GO:0065007 (1771) biological regulation
15	9				11			60	14	28		9	6	19	75	12	5	11	82	GO:0009058 (1141) biosynthetic process
	34	42											65				59	67		GO:0007155 (126) cell adhesion
	6	11	56	16	11		62		15	38	68	5	8	10	89	6	6	6		GO:0007154 (883) cell communication
	7	16	67	39	7			54	8	15	57	15	10	10	73		7	12	72	GO:0007049 (413) cell cycle
	34	31							22	36		19	12	27		35	30	30	85	GO:0051301 (108) cell division
52	38	54						80	52	59		19	16	35		23	85			GO:0008037 (42) cell recognition
39	12	17							28	17		17	8	18	82		12	13	69	GO:0044085 (411) cellular component biogenesis
15	6	14		36	12	35	69	53	7	13	65	5	8	6	71	5	6	6	65	GO:0016043 (1285) cellular component organization
9	7	19	81	20	12		64	61	6	30		7	10	15		7	7	11	77	GO:0048869 (776) cellular developmental process
	17	21			12			63	7	25	69	16	9	13		10	14	7	75	GO:0051641 (388) cellular localization
												19	17	73		41				GO:0007623 (46) circadian rhythm
36								59				8	46			20				GO:0016265 (161) death
	75											17	68	78		28	31			GO:0048589 (57) developmental growth
8	6	18		16	12		73	63	5	28		6	8	14		6	6	10	75	GO:0032502 (1367) developmental process
	14	18			44			83	15	13	65	52	6	14	52		11	10	67	GO:0051234 (1083) establishment of localization
	72		89						79			11	61	79		25	26			GO:0040007 (118) growth
													36	26			24	30		GO:0006955 (97) immune response
	9	17			43			83	15	17		9	6	19	70	8	10	14	67	GO:0051179 (1332) localization
41	13	29	79				66		48	51		9	11	41		14	26	23		GO:0051674 (232) localization of cell
	14				14				10	53		12	13	18		9	10	26		GO:0040011 (175) locomotion
	7	17			14			42	7	25		17	10	8		19	15	10	80	GO:0033036 (304) macromolecule localization
	8	19			15				9	15	61	11	12	13	77		9	8	69	GO:0007017 (289) microtubule-based process
												22	12			25	15	26		GO:0051704 (154) multi-organism process
12	6	18		16	12			60	6	29		7	8	10		6	7	6	75	GO:0007275 (1168) multicellular organismal development
16	6	17		16	11				6	28		6	7	9	91	6	6	6		GO:0032501 (1477) multicellular organismal process
												18	78			18	93			GO:0035265 (32) organ growth
66				57				37				22	95			69	57	63		GO:0055114 (315) oxidation reduction
													64	64			80			GO:0043473 (42) pigmentation
													65	65			82			GO:0048066 (41) pigmentation during development
15	10	23		15	11		59	32	6	28		5	7	9	88	5	5	10	75	GO:0050789 (1623) regulation of biological process
24	12	17			13	41		57	8	16		6	9	18		11	7	7		GO:0065008 (426) regulation of biological quality
21	13			25	13				7	32	57	8	10	21		7	8	21		GO:0000003 (437) reproduction
21	13			25	13				7	32	57	8	10	21		7	8	21		GO:0022414 (437) reproductive process
			98						10			9	14	16			27	32		GO:0009628 (131) response to abiotic stimulus
													29	28			22	29		GO:0009607 (85) response to biotic stimulus
					21							9	11			12	28	40		GO:0042221 (221) response to chemical stimulus
												12	15			13	15			GO:0009605 (129) response to external stimulus
					22				6			6	7			9	6			GO:0050896 (724) response to stimulus
												19	17	73		41				GO:0048511 (46) rhythmic process
22	13				12				17	31	58	8	10			6	7	21		GO:0019953 (380) sexual reproduction
	7		60		11				27			11	10	17		7	7	7		GO:0003008 (392) system process

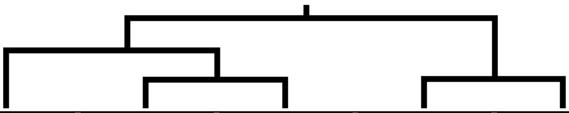

D. melanogast.				D. simulans				D. sechellia				D. yakuba				D. erecta				
dS	dN	ω	Δω	dS	dN	ω	Δω	dS	dN	ω	Δω	dS	dN	ω	Δω	dS	dN	ω	Δω	
	48	50							16	46		20	20	18		20	38	44		GO:0030534 (54) adult behavior
16	6	19	56	20	13		60		6	45		7	8	15	92	6	7	11		GO:0009653 (877) anatomical structure morphogenesis
28	14	33			20							10	9	37		8	9	26		GO:0048736 (164) appendage development
									59			34	65			29	40	34		GO:0008356 (44) asymmetric cell division
40	61				64			53	66	77		45	17	29		22	15	24		GO:0005975 (259) carbohydrate metabolic process
	8	18	70		7			61	9	16	59	6	12	12	77		8	13	74	GO:0022402 (340) cell cycle process
35								58	76			8	46			20				GO:0008219 (160) cell death
10	7	27		16	13		63	61	7	30		8	10	15		7	7	11	74	GO:0030154 (685) cell differentiation
	51	62					93					44	24	65			55	55		GO:0034330 (29) cell junction organization
41	13	29	76				66		49	46		10	11	41	93	14	27	23		GO:0006928 (229) cell motion
	10	21	65	30	14				11	25		16	13	16	87	8	9	16	83	GO:0007267 (156) cell-cell signaling
16	10				11			43	6	25		9	5	14	76	13	5	11		GO:0044249 (1086) cellular biosynthetic process
	11	22							28	17		16	8	19	76	78	13	14	72	GO:0022607 (376) cellular component assembly
			74		30				27			15	18	19		24	15			GO:0007635 (66) chemosensory behavior
	66												59	66						GO:0042335 (42) cuticle development
	40	38							14	46			14	34			31	44		GO:0000910 (47) cytokinesis
												59	31	24			18	25		GO:0006952 (100) defense response
26	35				14							16	9	68		11	13	22		GO:0009790 (312) embryonic development
	17	22			12				8	25		15	9	13	96		15	8	79	GO:0051649 (358) establishment of localization in cell
	19	35					86		38	26		19	13	24		42	20	20	91	GO:0043062 (73) extracellular structure organization
22	13			29	13				7	31	58	8	10			6	7			GO:0007276 (372) gamete generation
13	7	18		19	12			34	6	17		5	7	6	76	9	6	6		GO:0010467 (1000) gene expression
19	10	21		15	15	35			36	20	65	17	17	16	76	13	26			GO:0006091 (146) generation of precursor metabolites and energy
	46								9	29		13	50	46			35	43		GO:0042592 (162) homeostatic process
		36											40	45			27	45		GO:0002252 (22) immune effector process
	36		67		19				14	46		11	17	11		10	12	30		GO:0007626 (105) locomotory behavior
	23	24			46	43			32	19			15	20			16	16	73	GO:0043933 (220) macromolecular complex subunit organization
17	6				11			41	6	25		10	6	14	76	9	6	12		GO:0009059 (924) macromolecule biosynthetic process
			51										27	28		52				GO:0051321 (91) meiotic cell cycle
	13	20			51		67		9	26		16	8	20	59	16	8	9	76	GO:0016044 (259) membrane organization
	9	18	68		8			58	9	13	59	15	12	11	78		8	14	80	GO:0000278 (286) mitotic cell cycle
	77												68	81			59			GO:0022404 (22) molting cycle process
18	12			25	13			57	45			7	8	21		11	21	31		GO:0048519 (381) negative regulation of biological process
	7		60		19							11	10	17		7	15	7		GO:0050877 (371) neurological system process
53	39	39			36			82	53	60		17	17	36		24	87			GO:0008038 (41) neuron recognition
30													23	92	75	20	27	28		GO:0006139 (1066) nucleobase, nucleoside, nucleotide and nucleic acid metabo
38	6	15			13			55	8	15	55	5	10	7	74	6	7	13	66	GO:0006996 (659) organelle organization
25	13	32			13			68	18			11	9	31		11	8	24		GO:0007389 (290) pattern specification process
	11	21			23				16	28		59	13	22		6	35	43		GO:0006793 (381) phosphorus metabolic process
	36	36						65	67			25	24	28		31	32			GO:0048518 (249) positive regulation of biological process
22	22	26			15				17			8	9	17		7	8	23		GO:0009791 (303) post-embryonic development
	34	34							42	23		22	15	12		44	18	18	87	GO:0070271 (90) protein complex biogenesis
	8	21			14		65	59	19	26		18	11	9		19	16	11		GO:0008104 (242) protein localization
									50			35	70	70		50	65			GO:0032535 (20) regulation of cellular component size
15	10	17	79	18	11		59	36	5	36		5	6	9	89	5	10	10	75	GO:0050794 (1531) regulation of cellular process
26	14	28		61	13			56	17	32	56	7	10	17		8	7	17		GO:0050793 (318) regulation of developmental process
									75			12	67			34	78	84		GO:0040008 (73) regulation of growth
68									27	41		25	20	30		42	18	14		GO:0032879 (70) regulation of localization
18	11			45	12			35	6			5	7	20		10	11	20		GO:0019222 (739) regulation of metabolic process
30	14	33			21				9	39		14	10	18		14	25	74		GO:0051239 (210) regulation of multicellular organismal process
													29	22			22	29		GO:0051707 (79) response to other organism
												24	18	39		26	30	44		GO:0009314 (69) response to radiation
									12	39	67		12	73			24			GO:0006403 (79) RNA localization
13	7	14	64	12	13		60	61	6	29		7	9	6	92	7	8	7	92	GO:0048731 (939) system development
	14	18			45				15	13	65	52	6	15	52		11	10	67	GO:0006810 (1059) transport

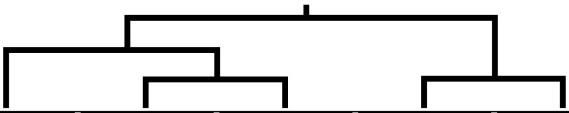

D. melanogast.				D. simulans				D. sechellia				D. yakuba				D. erecta				
dS	dN	ω	Δω	dS	dN	ω	Δω	dS	dN	ω	Δω	dS	dN	ω	Δω	dS	dN	ω	Δω	
		50							23	63		23	26	43		26	43	36		GO:0008344 (30) adult locomotory behavior
64	30	34			43				27	42			18	25		18	10	23		GO:0006040 (76) amino sugar metabolic process
					32				23	39		35	15	26			26	19		GO:0048532 (71) anatomical structure arrangement
34	14											12	12			12	23			GO:0048646 (248) anatomical structure formation involved in morphogenesis
28	14	38			20							10	9	44		8	9	26		GO:0035107 (163) appendage morphogenesis
34					26				23	35	63	31	12	20		22	20	27		GO:0009798 (115) axis specification
18	6	24		30	12			37	6	26		5	7	15		9	6	12		GO:0043284 (863) biopolymer biosynthetic process
									51				11	29		36	41	45	91	GO:0043412 (510) biopolymer modification
	8	19	72		7			64	8	16	60	6	11	11	78		8	13	74	GO:0022403 (301) cell cycle phase
11	7	26		21	13		85	62	7	30		8	10	16		7	8	11	70	GO:0048468 (602) cell development
27	15	33			13				9			19	9	70		15	8	46		GO:0045165 (186) cell fate commitment
	14						74		48	53		11	11			15	26			GO:0048870 (127) cell motility
	66	62					88					44	25	37			59	59		GO:0045216 (27) cell-cell junction organization
17	7	20	76	24	12		62		16	35		7	10	11	91	7	7	23	81	GO:0032989 (506) cellular component morphogenesis
	31	24			47				32	19			19	19			15	17	73	GO:0034621 (182) cellular macromolecular complex subunit organization
17	6				11			41	6	25		10	6	14	76	9	6	12		GO:0034645 (924) cellular macromolecule biosynthetic process
	24	67			16				31	38		30	10	16			21	10		GO:0034613 (130) cellular protein localization
91					58							60	27	34			50	51		GO:0034984 (70) cellular response to DNA damage stimulus
													54	54			45	54		GO:0050000 (11) chromosome localization
	25	42							24	31		15	11	16	14	22	14	19	46	GO:0050890 (226) cognition
18	10	20			7			63	10	13	56	7	15	12	78	8	11	7	70	GO:0007010 (353) cytoskeleton organization
	31	31		23	39	43			41	43			25	15		13				GO:0022900 (51) electron transport chain
31	68		65		17				58			15	15	34		10	12	17		GO:0009792 (132) embryonic development ending in birth or egg hatching
	16	30							49	50		18	35	51		14	10	53		GO:0048598 (128) embryonic morphogenesis
	10	23			17	44	67	55	22	31		18	12	11		12	20	13		GO:0045184 (174) establishment of protein localization
	15		80									15	17	53		26	17	60		GO:0007164 (45) establishment of tissue polarity
	25	22										21	12	26		14	17	16		GO:0035272 (112) exocrine system development
26	13				14				8	29	58	13	10	65		12	8	22		GO:0007292 (284) female gamete generation
80	13	23		27	27			45	13	37		17	16	31		25	33	17		GO:0003001 (80) generation of a signal involved in cell-cell signaling
	32	34		26					26	34	76		23			19				GO:0006818 (52) hydrogen transport
	24	38			19		70					9	9	38		14	9	25		GO:0048737 (162) imaginal disc-derived appendage development
27	22	26		23	15				17			8	9	17		7	8	24		GO:0002165 (288) instar larval or pupal development
	18	22			13				8	21		76	8	8			12	14		GO:0046907 (278) intracellular transport
	46	52							20	23		12	8			7	8	67		GO:0006811 (232) ion transport
43	18	43			28				31			21	31	46		28	21	40		GO:0060173 (32) limb development
	29	30			43	45			31	20			16	23			15	16	81	GO:0065003 (186) macromolecular complex assembly
	45								20	41			66			54	41	54		GO:0032984 (24) macromolecular complex disassembly
17		17	17	34							65	12	11							GO:0048232 (79) male gamete generation
	17	22			34		70		8	27		18	10	14	61	12	9	11	77	GO:0010324 (205) membrane invagination
26	22	45			16				8			9	10	17		7	9	24		GO:0007552 (253) metamorphosis
46	26								65	68		21	19	31		39	63			GO:0042692 (41) muscle cell differentiation
23	12			26	14			62	40			7	9	17		7	7	22		GO:0048523 (341) negative regulation of cellular process
33	17			68	16			73	41			10	12	36		10	27	57		GO:0051093 (103) negative regulation of developmental process
24								64	46			18	9	86		21	32			GO:0009892 (202) negative regulation of metabolic process
18	7	28	66	59	12		62		8	35		7	9	16	81	7	8	12	78	GO:0007399 (495) nervous system development
75	12	30		33	30				12	41		23	13	28		22	30	15		GO:0006836 (89) neurotransmitter transport
	57	70							29	23	63		35							GO:0055086 (151) nucleobase, nucleoside and nucleotide metabolic process
			74	70	30							12	16			22	12			GO:0042048 (62) olfactory behavior
	43	41							52	53		24	10	35		46	39	40		GO:0007424 (123) open tracheal system development
17	6	26		16	13		61	61	6	34		7	9	16		7	8	7		GO:0048513 (753) organ development
	86												46	46			60	46		GO:0043043 (15) peptide biosynthetic process
	11	21			23				16	28		59	13	22		6	35	43		GO:0006796 (381) phosphate metabolic process
64	30	34			41				28	35			17	24		17	9	22		GO:0005976 (81) polysaccharide metabolic process
	36							68	71			25	23	36		35	32			GO:0048522 (226) positive regulation of cellular process
26	22	35		23	16				8			8	10	18		7	9	24		GO:0009886 (248) post-embryonic morphogenesis
	85												42	50			64	50		GO:0002440 (14) production of molecular mediator of immune response
	64							60				36	46			12				GO:0012501 (155) programmed cell death
25	13	32			13			68	18			11	9	27		16	24	24		GO:0003002 (274) regionalization
	39	34										26	26	39		47	34	52		GO:0032970 (23) regulation of actin filament-based process
30	19	23		33	16			60	21	36		9	14	14		10	9	14		GO:0022603 (171) regulation of anatomical structure morphogenesis
20					11							14	72			9	42	82		GO:0009889 (553) regulation of biosynthetic process
	56						80		58			24	49	64		11	28	38		GO:0010646 (221) regulation of cell communication
								72	79			53	67			25				GO:0010941 (88) regulation of cell death
34	19	25			17			69	10	32		16	13	23		16	16	15		GO:0045595 (137) regulation of cell differentiation
29	17	30			15			67	22	31		8	12	22		24	15	17		GO:0051128 (186) regulation of cellular component organization
	60				21				19	43	78	43	14	41			29	29		GO:0060341 (41) regulation of cellular localization
17	61				11			42	70			10	10	81		9	21	20		GO:0031323 (673) regulation of cellular metabolic process
													42	42			31	42		GO:0031347 (19) regulation of defense response
												70	88	81			88			GO:0048638 (27) regulation of developmental growth
18	10			37	11			35	6			11	6	78		10	11	20		GO:0060255 (704) regulation of macromolecule metabolic process
												27	20	40		28	33	45		GO:0009416 (59) response to light stimulus
78	12	27		30	27				11	41		15	14	19		15	11	15		GO:0046903 (99) secretion
	11	14		23	11		61		16	60	68	5	7	15		6	7	24		GO:0007165 (741) signal transduction
									27			20	47			27	47	68		GO:0048863 (44) stem cell differentiation
	36								40	32		22	28	28		46	22	24	93	GO:0050808 (49) synapse organization
	11	17	67		23		69		11	30		17	14	17	87	9	10	17	83	GO:0019226 (140) transmission of nerve impulse
	33	45										33	20	29			33	25		GO:0016050 (24) vesicle organization
71	13	20			33		64		18	35		16	8	12	63	17	7	10		GO:0016192 (288) vesicle-mediated transport

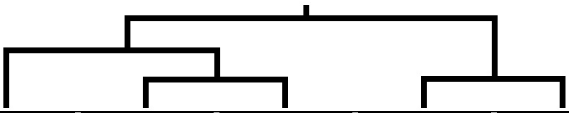

D. melanogast.				D. simulans				D. sechellia				D. yakuba				D. erecta				
dS	dN	ω	Δω	dS	dN	ω	Δω	dS	dN	ω	Δω	dS	dN	ω	Δω	dS	dN	ω	Δω	
38	10	22							20	23		17	18	26		12	13	16		GO:0030036 (121) actin cytoskeleton organization
35					25				21	35		18	13	71			86	27		GO:0009952 (98) anterior/posterior pattern formation
	85												42	50			64	50		GO:0002775 (14) antimicrobial peptide production
								71				56				19				GO:0006915 (100) apoptosis
	52	52											64	84			68	64		GO:0006576 (25) biogenic amine metabolic process
												29	79			15	37			GO:0001709 (91) cell fate determination
	54								76			31	52			23	68			GO:0001708 (51) cell fate specification
	15	47					75		49	53		11	12			15	27			GO:0016477 (125) cell migration
24	14	20	70	32	12		63		16	36		7	9	16	91	6	7	11	81	GO:0000902 (470) cell morphogenesis
39	15	32	77		21		67		39	39		7	10	25	93	13	28	25		GO:0032990 (228) cell part morphogenesis
	49	71			13				16			6	8	60		6	7	50		GO:0007166 (407) cell surface receptor linked signal transduction
18	6	24		30	12			37	6	26		5	7	15		9	6	12		GO:0034961 (863) cellular biopolymer biosynthetic process
	30	24			35	48			32	20			21	22			15	18	83	GO:0034622 (155) cellular macromolecular complex assembly
	45								20	41			66			54	41	54		GO:0034623 (24) cellular macromolecular complex disassembly
41										40		30	16	30		9	36	54		GO:0007417 (143) central nervous system development
	34	36						92	40	33		91	89	82			37	31	88	GO:0030705 (69) cytoskeleton-dependent intracellular transport
												50	35	50			57	64		GO:0050832 (14) defense response to fungus
	37	40										54	28	41			44	46		GO:0006259 (154) DNA metabolic process
31	69		66		17				58			16	15	34		10	12	17		GO:0001700 (129) embryonic development via the syncytial blastoderm
	17	22			34		70		8	27		18	10	14	61	12	9	11	77	GO:0006897 (205) endocytosis
													54	54			45	54		GO:0051303 (11) establishment of chromosome localization
									33			41	41	75		33	58			GO:0007629 (12) flight behavior
	46	51			33							56	17			20	84			GO:0048859 (39) formation of anatomical boundary
27				27	23				45		65	18	14	22		22	20	30		GO:0007281 (140) germ cell development
	26	22			15							21	12	10		15	18	17		GO:0048732 (129) gland development
64	30	34			43				28	36			18	25		17	10	24		GO:0006041 (75) glucosamine metabolic process
	24	27										16	18			14				GO:0007507 (54) heart development
20	12	35			15		69		8			9	11	22		7	9	24		GO:0007444 (287) imaginal disc development
	24	37			19							9	9	37		14	9	26		GO:0035114 (161) imaginal disc-derived appendage morphogenesis
26	22	36		31	16				8			9	10	18		7	9	24		GO:0048707 (244) instar larval or pupal morphogenesis
	12	19			13		71		19	41		16	8	18		7	8	14		GO:0007242 (291) intracellular signaling cascade
	14				21				38	42		21	21	15		17	19			GO:0007611 (57) learning or memory
43	18	43			28				31			21	31	46		28	21	40		GO:0035108 (32) limb morphogenesis
	8	19	71		16				9	21	60	7	12	11	78		9	13	71	GO:0000279 (276) M phase
	31	43							50			43	25	37		68	50	31		GO:0006900 (16) membrane budding
38	10	20			9				11	14	60	13	14	13	79	12	11	17	70	GO:0000226 (222) microtubule cytoskeleton organization
	33								53			33	40	53		26	26			GO:0055001 (15) muscle cell development
41	19	33			15		71		28	40		16	12	20		25	20	15	76	GO:0007517 (139) muscle development
30												18	14	86		19	54			GO:0009890 (143) negative regulation of biosynthetic process
					21				36	43		18	14	56		20	40			GO:0045596 (55) negative regulation of cell differentiation
24								67				18	9	87		16	24	24		GO:0031324 (175) negative regulation of cellular metabolic process
24								65	66			18	8	85		16				GO:0010605 (192) negative regulation of macromolecule metabolic process
22	7	31	79	40	13		64		8	33		8	10	25		7	8	16	79	GO:0022008 (366) neurogenesis
					19				14	48	69		21				64			GO:0051169 (56) nuclear transport
		73							31	24	64		36	34						GO:0006753 (135) nucleoside phosphate metabolic process
26	13				13				8	29	58	13	10	66		12	8	22		GO:0048477 (281) oogenesis
22	13	27	64	22	15		67		8			8	9	22		7	7	24		GO:0009887 (361) organ morphogenesis
	12	21			25				18	26		59	14	18	94	7	30	43	95	GO:0016310 (310) phosphorylation
	34	34							42	23		22	15	12		44	18	18	87	GO:0006461 (90) protein complex assembly
	47								21	43			69			34	43	39		GO:0043241 (23) protein complex disassembly
	10	23			17	45		61	23	32		18	12	11		16	20	13		GO:0015031 (169) protein transport
35	20	31			17			63	12	35		17	14	26		15	23	24		GO:0060284 (97) regulation of cell development
												56				68				GO:0010453 (16) regulation of cell fate commitment
20					11							14	72			9	42	82		GO:0031326 (553) regulation of cellular biosynthetic process
17				41	11			39				10	59	81		9	20	29		GO:0010468 (604) regulation of gene expression
													91	91			65	60		GO:0043087 (23) regulation of GTPase activity
	85												42	50			64	50		GO:0002697 (14) regulation of immune effector process
26												14	69	93		9	42	82		GO:0010556 (534) regulation of macromolecule biosynthetic process
67	21			72	18			67	25	47		18	12	25		21	35	77		GO:0051960 (70) regulation of nervous system development
20	75				10							14	63			10	25	30		GO:0019219 (549) regulation of nucleobase, nucleoside, nucleotide and nucleic
	80							73				52	62			25				GO:0043067 (87) regulation of programmed cell death
11	9				27			48	12	25		16	14	10	81	13	10	27		GO:0051246 (143) regulation of protein metabolic process
	56						80		65			32	49	64		21	33	37		GO:0009966 (206) regulation of signal transduction
	32	32		24	40	44			40	42			26	16		14				GO:0022904 (49) respiratory electron transport chain
32								59				24	19	28	90	13	15	15		GO:0016070 (687) RNA metabolic process
81	13	28		29	27				12	42		24	13	19		16	12	15		GO:0032940 (91) secretion by cell
24	14	32		23	13		68		10			16	15	28		7	14	24		GO:0007423 (257) sensory organ development
	27	48							26	32		12	13	19	17	16	17	22	39	GO:0007600 (173) sensory perception
17		17	17	34							65	12	11							GO:0007283 (79) spermatogenesis
	11	23	67	30	23		68		11	30		18	14	18	87	9	10	17		GO:0007268 (138) synaptic transmission
	14	22			27			50	16	45		17	14	30		16	13	19		GO:0048489 (68) synaptic vesicle transport
									80			29	32	41		54	38	38		GO:0007416 (31) synaptogenesis
	14	28			13				46			8	10	17	92	14	9	14		GO:0009888 (281) tissue development
27	31	34							31	38	78		23			17				GO:0034220 (47) transmembrane ion transport

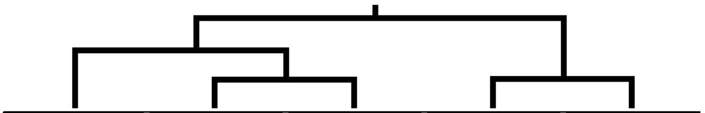

D. melanogast.				D. simulans				D. sechellia				D. yakuba				D. erecta				
dS	dN	ω	Δω	dS	dN	ω	Δω	dS	dN	ω	Δω	dS	dN	ω	Δω	dS	dN	ω	Δω	
43	20	24			42				12	25		16	15	24		11	12	32		GO:0007015 (78) actin filament organization
23	23								30	53		26	30	43		23	20			GO:0031032 (30) actomyosin structure organization
													44	40			44	44		GO:0019731 (20) antibacterial humoral response
	85												42	50			64	50		GO:0002777 (14) antimicrobial peptide biosynthetic process
69	38											53	61	69		38	53	76		GO:0035285 (13) appendage segmentation
									13	49		34	11	35		16	39	58		GO:0007420 (79) brain development
44	63								60			31	39	44		34	36	34		GO:0022416 (38) bristle development
39	15	32	69		21		68		40	39		7	9	25	93	13	13	26		GO:0000904 (236) cell morphogenesis involved in differentiation
40	13	18	68		24		68		38	39		7	9	22		12	26	23		GO:0030030 (289) cell projection organization
		71											52	66			52	66		GO:0007043 (21) cell-cell junction assembly
	24	33			45	56			50	40		98	14	29			22	26		GO:0043623 (57) cellular protein complex assembly
	47								21	43			69			34	43	39		GO:0043624 (23) cellular protein complex disassembly
	54	90							81	72			36	100						GO:0046545 (11) development of primary female sexual characteristics
	60	64							71	75			35	46		71	57			GO:0006310 (28) DNA recombination
96	54											59	29	35			37	62		GO:0006281 (62) DNA repair
	63	56											69	61			38	40		GO:0007398 (55) ectoderm development
	17	30	69		15		69		21	41		9	14	22	96	9	11	18		GO:0060429 (139) epithelium development
	23		75									13	13	18	100	21	39	18		GO:0007163 (76) establishment or maintenance of cell polarity
	55	57											53	38			25	23		GO:0006887 (47) exocytosis
25	15	33		28	14			57	9			12	16	32		7	14	24		GO:0001654 (204) eye development
21	15	31	71	37	13		64		8	32		8	10	24		7	8	25		GO:0048699 (353) generation of neurons
	86											59	81	81		22	95			GO:0007446 (22) imaginal disc growth
27	34	37			16		69					9	10	24		13	26	24		GO:0007560 (198) imaginal disc morphogenesis
34	13							81				40	18			17	30			GO:0007447 (69) imaginal disc pattern formation
46	69	65			26				34			88	30	73		30	23	46		GO:0035109 (26) imaginal disc-derived limb morphogenesis
	25	68			17				32	39		31	10	17			21	10		GO:0006886 (128) intracellular protein transport
					35							30	20			30	17			GO:0007612 (40) learning
43	18	43			28				31			21	31	46		28	21	40		GO:0035110 (32) leg morphogenesis
			53									55	20	25		46				GO:0051327 (86) M phase of meiotic cell cycle
													54	54			45	54		GO:0051310 (11) metaphase plate congression
		65							36	34			89	86			36	31	87	GO:0007018 (66) microtubule-based movement
									20	33	63	19	10			12	10			GO:0015672 (121) monovalent inorganic cation transport
	15								36	38	64		21	16	84		15	17	82	GO:0016071 (139) mRNA metabolic process
48	67								55	59		26	32	83		46	32	32		GO:0048747 (49) muscle fiber development
64	30	34			43				28	36			18	25		17	10	24		GO:0006044 (75) N-acetylglucosamine metabolic process
58												75				75				GO:0010454 (12) negative regulation of cell fate commitment
30												18	14	86		19	54			GO:0031327 (143) negative regulation of cellular biosynthetic process
32								76	70			23	75	87		26	86			GO:0010629 (140) negative regulation of gene expression
30												18	14	86		19	54			GO:0010558 (142) negative regulation of macromolecule biosynthetic process
33												24	75	88		23	55			GO:0045934 (127) negative regulation of nucleobase, nucleoside, nucleotide an
	33	89										35	69	43		30	66			GO:0043069 (39) negative regulation of programmed cell death
					19				14	48	69		21				64			GO:0006913 (56) nucleocytoplasmic transport
		73							31	24	64		36	34						GO:0009117 (135) nucleotide metabolic process
					28				49	36		16	14	25		25	25	19		GO:0009994 (83) oocyte differentiation
44												27	10			19				GO:0030707 (131) ovarian follicle cell development
												38	26	42			42	38		GO:0007300 (26) ovarian nurse cell to oocyte transport
16	11	26		20	16	39			25	22	71	20	18	18		15				GO:0006119 (96) oxidative phosphorylation
	19	25					77		31	28			32	19	63		19	26		GO:0006909 (151) phagocytosis
	45	40			30				25	51			20	25		43	40	45		GO:0007243 (62) protein kinase cascade
									47				12	29		36	34	46	81	GO:0006464 (484) protein modification process
												79	40	33		92	33	28		GO:0065004 (77) protein-DNA complex assembly
	20	23		89	58	69		47	18	35		20	6	12		16	4	11	5	GO:0006508 (427) proteolysis
	39	34										26	26	39		47	34	52		GO:0032956 (23) regulation of actin cytoskeleton organization
60												73				73				GO:0042659 (15) regulation of cell fate specification
30	19	33			17				21	38		9	12	23		10	10	17		GO:0022604 (139) regulation of cell morphogenesis
34									34	34		16	12	54		30	32	41		GO:0032268 (114) regulation of cellular protein metabolic process
	13	25		36	26				13	36		24	14	31		24	34	18		GO:0001505 (83) regulation of neurotransmitter levels
	85												42	50			64	50		GO:0002700 (14) regulation of production of molecular mediator of immune resp
25								43				10	54			10	21	21		GO:0051252 (437) regulation of RNA metabolic process
	25								41	41		32	25	48		38	32	22		GO:0050803 (31) regulation of synapse structure and activity
24												19	54			15	22	22		GO:0032774 (442) RNA biosynthetic process
	13							64	55				27	20	89		19	20	79	GO:0006396 (187) RNA processing
	25	22										21	12	26		14	17	16		GO:0007431 (112) salivary gland development
	30	29			55		33		30	32		14	11	13	21	21	13	21	19	GO:0007606 (123) sensory perception of chemical stimulus
	11	21			24				24			19	13	23	63	11	13	19	79	GO:0007264 (136) small GTPase mediated signal transduction
43	11	22			10	46			11	15	61	15	16	15	80		11	17	73	GO:0007051 (171) spindle organization
	44	50	81		42				50	60			15	47		31	15	42		GO:0031098 (38) stress-activated protein kinase signaling pathway
44	21				17				35	32		11	17	26		29	20	29		GO:0014706 (79) striated muscle development
	35	50										50	28	42		57	57	35		GO:0070142 (14) synaptic vesicle budding
31	25	34		43	34			56	25			28	28	28		28	28	28		GO:0048488 (32) synaptic vesicle endocytosis
	17	31	66				69		39	41		15	12	19	94	14	10	16	79	GO:0048729 (173) tissue morphogenesis
24	62											18	69			14	29	73		GO:0006350 (541) transcription
7	12	20		11	9	44		13	10	26	64	11	14	12		8	14	11		GO:0006412 (264) translation
	46	61			46				53			46	53	38		69	53	30		GO:0006901 (13) vesicle coating
45	33	46			17		66		49			8	9	41		17	26	24		GO:0035220 (186) wing disc development
	50	54					75					39	14	54		13	14	63		GO:0016055 (61) Wnt receptor signaling pathway

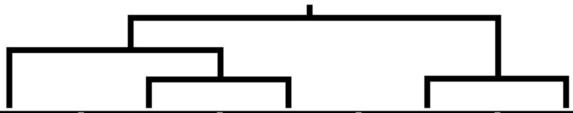

D. melanogast.				D. simulans				D. sechellia				D. yakuba				D. erecta				
dS	dN	ω	Δω	dS	dN	ω	Δω	dS	dN	ω	Δω	dS	dN	ω	Δω	dS	dN	ω	Δω	
52	44	44							48	40		40	24	36		36	36	40		GO:0008154 (25) actin polymerization or depolymerization
		71											52	66			52	66		GO:0043297 (21) apical junction assembly
	31	31		23	40	46			42	44			31	17		27				GO:0042773 (47) ATP synthesis coupled electron transport
39	15	32	77		21		67		39	39		7	10	25	93	13	28	25		GO:0048858 (228) cell projection morphogenesis
	31	37			47		41		30	38			19	19		18	11	25		GO:0006030 (70) chitin metabolic process
26	16	33		44	15			60	10			13	8	40		8	8	25		GO:0048749 (192) compound eye development
	84												61	76		61	46	61		GO:0050907 (13) detection of chemical stimulus involved in sensory perception
													30	65			55	65		GO:0035088 (20) establishment or maintenance of apical/basal cell polarity
	14	35		29	14			55	9			8	10	31		8	14	24		GO:0048592 (161) eye morphogenesis
69	38											53	61	69		38	53	76		GO:0035286 (13) leg segmentation
	78	46	76		36				40	61			21	27		27	31			GO:0000165 (47) MAPKKK cascade
			53									55	20	25		46				GO:0007126 (86) meiosis
	12	23			11	45			12	16	61	8	16	16	81	9	11	18	74	GO:0007052 (155) mitotic spindle organization
	17	30	69		15		69		21	41		9	14	22	96	9	11	18		GO:0002009 (139) morphogenesis of an epithelium
	15								32	29	72		23	18	83	80	17	18	78	GO:0006397 (123) mRNA processing
58												75				75				GO:0009996 (12) negative regulation of cell fate specification
22	15	31	75	38	13		64		18	33		8	10	24		7	8	12		GO:0030182 (317) neuron differentiation
80	14	24		27	28			67	14	36		16	15	18		25	11	18		GO:0007269 (77) neurotransmitter secretion
									27	36	72		19	30		12				GO:0009141 (66) nucleoside triphosphate metabolic process
									34	34	64		41	67						GO:0009165 (113) nucleotide biosynthetic process
					34							31	20			28	17			GO:0008355 (35) olfactory learning
				38	33				23	38		34	15	26			26	19		GO:0048599 (72) oocyte development
	47	37							14	52		25	16			33	33			GO:0007297 (48) ovarian follicle cell migration
	20	20					77		31	28			20	20	64	82	27	26		GO:0006911 (145) phagocytosis, engulfment
	11				12				7	51			13	21		22	30	43	82	GO:0043687 (388) post-translational protein modification
	56	56							25	56			50			43	56	50		GO:0051261 (16) protein depolymerization
	37	37			51	70			66	37		33	25	22		44	25	25		GO:0051258 (27) protein polymerization
	32	34		26					26	34	76		23			19				GO:0015992 (52) proton transport
									22	27	72									GO:0006163 (83) purine nucleotide metabolic process
												97	60	87			53	60		GO:0007265 (71) Ras protein signal transduction
58									52			41	70			58	52			GO:0030832 (17) regulation of actin filament length
	85												42	50			64	50		GO:0002784 (14) regulation of antimicrobial peptide production
55	23				38		82					20	20	32		20	26	52		GO:0031344 (34) regulation of cell projection organization
52	20	34		72	18				44			41	30	37		27	34	34		GO:0008360 (70) regulation of cell shape
	23			74	21			76	27	49		18	12	27		20	32	58		GO:0050767 (55) regulation of neurogenesis
		53										56	86							GO:0010627 (30) regulation of protein kinase cascade
										88		85	61	80			44	58		GO:0051056 (63) regulation of small GTPase mediated signal transduction
	42								66	42		42	47	47		61	52	33		GO:0050807 (21) regulation of synapse organization
25												19	73			15	43	73		GO:0045449 (483) regulation of transcription
									24	29	69									GO:0009259 (86) ribonucleotide metabolic process
	19	32							29	37			25	19	83	73	25	20		GO:0008380 (97) RNA splicing
60	51	55							44	69		55	25	39		27	27	46		GO:0007608 (43) sensory perception of smell
	36	44			63				34	40			20	20	28		30	28	28	GO:0050909 (49) sensory perception of taste
	18				18				40	43		18	13	81		44	18			GO:0007519 (65) skeletal muscle development
										80		45	52	69			42	69		GO:0006814 (42) sodium ion transport
24	26	35		22	19	42		59	22	36	71	19	35	28	98	14	22	15	89	GO:0051231 (57) spindle elongation
	31								50			31	37	50		25	25			GO:0051146 (16) striated muscle cell differentiation
	35	50										50	28	42		57	57	35		GO:0016185 (14) synaptic vesicle budding from presynaptic membrane
24												19	54			15	22	22		GO:0006351 (441) transcription, DNA-dependent
	35	38			19		71					8	9	44		18	26	25		GO:0007472 (146) wing disc morphogenesis
