# Supplementary material for: Natural Selection on Functional Modules, a Genome-Wide Analysis
Source: PLoS Comput Biol. 2011 Mar 3;7(3):e1001093. doi: 10.1371/journal.pcbi.1001093 (PMC3048381; doi:10.1371/journal.pcbi.1001093)
Supplement: Figure S3 — Complete list of significant results of GSSA for KEGG pathways in mammals species. GSSA (5% FDR) results (82 KEGG pathways) for dS, dN, ω & Δω in mammals species. (1.54 MB PDF) [file pcbi.1001093.s003.pdf]

H. sapiens				P. troglodytes				M. musculus				R. norvegicus				
dN	dS	ω	Δω	dN	dS	ω	Δω	dN	dS	ω	Δω	dN	dS	ω	Δω	
	36	65			41	54	84		45	45	95		58	52		hsa05221 (46) Acute myeloid leukemia
	73	64			66	66			31	31			33	15	89	hsa04520 (57) Adherens junction
			54						45	45			54	54		hsa05330 (11) Allograft rejection
	79		16				14							8	14	hsa05010 (113) Alzheimers disease
	29	63					17		51	26			51	41	36	hsa04612 (41) Antigen processing and presentation
				82					50	58	90		33	66		hsa05412 (62) Arrhythmogenic right ventricular cardiomyopathy (ARVC)
			58				50		50	41			58	41		hsa05310 (12) Asthma
	26	50	30		38	50	23		26	34		69	50	53	46	hsa05320 (26) Autoimmune thyroid disease
	71	64							32	27	84		22	27	73	hsa04360 (109) Axon guidance
									65	72			72	48		hsa03410 (29) Base excision repair
29	77	62	78						35	38				59	89	hsa04020 (131) Calcium signaling pathway
	59			46	22	39							51	28	70	hsa04110 (100) Cell cycle
	65	63			62				41	43			25	30		hsa04062 (123) Chemokine signaling pathway
									92	84			100	92		hsa00532 (13) Chondroitin sulfate biosynthesis
	60	65			25	46	81		45	45	91		43	50		hsa05220 (60) Chronic myeloid leukemia
	36	40			21	43	69		44	47	98		30	29	98	hsa05210 (65) Colorectal cancer
	27	69			39	62		88	30	23	20	74	30	25	32	hsa04610 (43) Complement and coagulation cascades
	17	29	13		24	37	14		12	12	14		22	18	13	hsa04060 (174) Cytokine-cytokine receptor interaction
	26		23		21		21		50	30		57	38	35	30	hsa04623 (42) Cytosolic DNA-sensing pathway
	80		91				95		38	44	92		35	34	75	hsa05414 (70) Dilated cardiomyopathy
	53		32		50		39		35	28			60	57	28	hsa00982 (28) Drug metabolism - cytochrome P450
79	41	67		77	57	80			63	67			77	72		hsa04512 (68) ECM-receptor interaction
	70	65				68			26	26	78		35	30		hsa04144 (140) Endocytosis
	72	64			71	67			47	42			28	48		hsa04012 (70) ErbB signaling pathway
	75								46	51			57	11		hsa04666 (76) Fc gamma R-mediated phagocytosis
		84	86	55			91		43	56	88		67	82	84	hsa04510 (153) Focal adhesion
	72	67			83	73			19	20	92		27	29	91	hsa04540 (68) Gap junction
	81								45	59			34	27		hsa05214 (44) Glioma
	56								43	50			50	50	37	hsa00531 (16) Glycosaminoglycan degradation
	79	68							36	46			36	34		hsa04912 (73) GnRH signaling pathway
			60				50		50	70	60		60	30		hsa05332 (10) Graft-versus-host disease
	87		95		46		95		39	36			34	43		hsa04340 (41) Hedgehog signaling pathway
	52	64	15			47	15		13	13	18		24	16		hsa04640 (53) Hematopoietic cell lineage
									58	52			70	76		hsa00534 (17) Heparan sulfate biosynthesis
	76	63			67	53	76		36	36			30	35		hsa04910 (104) Insulin signaling pathway
			9				11		10	16	10		25	19	14	hsa04630 (111) Jak-STAT signaling pathway
	79	62			65	60	91		43	52			32	11		hsa04670 (78) Leukocyte transendothelial migration
									46	46	46		73	73	46	hsa00591 (15) Linoleic acid metabolism
	50	60							33	39	94		13	32		hsa04730 (53) Long-term depression
	65	63					93		22	28	95		16	18	85	hsa04720 (49) Long-term potentiation
	50	57	91		14	58	90		8	17	82		14	8	80	hsa04010 (210) MAPK signaling pathway
	91		92				91		27	20			28	13		hsa04916 (69) Melanogenesis
	65				28	48		48	23	25		17	15	17		hsa05218 (52) Melanoma
	54		45				41		37	37			54	50		hsa00980 (24) Metabolism of xenobiotics by cytochrome P450
			22		32		14		15	22			29	27	21	hsa04650 (84) Natural killer cell mediated cytotoxicity
	57	36			65	64			41	41			17			hsa04722 (92) Neurotrophin signaling pathway
							36		54	63			81	81	72	hsa03450 (11) Non-homologous end-joining
	37	69				72	86		55	58			44	48	100	hsa05223 (43) Non-small cell lung cancer
											66		89			hsa03420 (39) Nucleotide excision repair
	25	19	19		12	30	28		44	44			39	36	38	hsa04740 (136) Olfactory transduction
	32	38		60	29	40			14	25			14	14		hsa04114 (84) Oocyte meiosis
							50		58	75			50	58	75	hsa00511 (12) Other glycan degradation
			14	41			12				15		62	9	17	hsa00190 (82) Oxidative phosphorylation
	29				24	36	77		43	45			31	31	100	hsa05212 (57) Pancreatic cancer
			14	40			11	56	65	52	15		55	11	11	hsa05012 (76) Parkinsons disease
	64	61							29	38			38	64		hsa05130 (34) Pathogenic Escherichia coli infection
	65	57	85		15	30	61		17	25	84		23	24	90	hsa05200 (245) Pathways in cancer
		70							44	50			67	34		hsa04146 (61) Peroxisome
			52			36	42			84						hsa00860 (19) Porphyrin and chlorophyll metabolism
			54						45	45	45		54	54		hsa00120 (11) Primary bile acid biosynthesis
			32		60				36	31			36	31	44	hsa05340 (25) Primary immunodeficiency
	33	41			33	41	91		43	45			35	27	77	hsa04914 (62) Progesterone-mediated oocyte maturation
	65				40	45	80		37	37			31	25		hsa03050 (35) Proteasome
	57	63							63	63			42	42		hsa03060 (19) Protein export
										87			83	82		hsa00230 (124) Purine metabolism
	62	65			93	63	88		15	27	87	8	25	24	89	hsa04810 (158) Regulation of actin cytoskeleton
	28				50		17		46	46		71	46	46	39	hsa04140 (28) Regulation of autophagy
	59	66			70				50	56			31	29		hsa05211 (57) Renal cell carcinoma
						61	76									hsa00740 (13) Riboflavin metabolism
50	76			57	76	69			26	38			38	23		hsa03010 (26) Ribosome
							25		34	32			30	27	21	hsa04622 (55) RIG-I-like receptor signaling pathway
								94					47	35		hsa03020 (17) RNA polymerase
	20	33	80		50	33	79		23	37	81		16	15	72	hsa03040 (86) Spliceosome
			40				28		31	36	52		52	40	32	hsa00140 (25) Steroid hormone biosynthesis
			42						21	27	24		24	24		hsa05322 (33) Systemic lupus erythematosus
	57	59	93		59				36	42			33	37		hsa04350 (66) TGF-beta signaling pathway
	71	59			67	59			21	36	93		27	12		hsa04530 (91) Tight junction
							11		35	19			22	22	17	hsa04620 (70) Toll-like receptor signaling pathway
									74	80		91	74	82		hsa00380 (35) Tryptophan metabolism
81	32	39		63	18	32			13	22			12	11	70	hsa04120 (106) Ubiquitin mediated proteolysis
									35	19			16	16		hsa05110 (42) Vibrio cholerae infection
	30	55	86		13	38	90		18	19	78		12	16	95	hsa04310 (111) Wnt signaling pathway
