# Supplementary figures and images for: Natural Selection on Functional Modules, a Genome-Wide Analysis
Source: PLoS Comput Biol. 2011 Mar 3;7(3):e1001093. doi: 10.1371/journal.pcbi.1001093 (PMC3048381; doi:10.1371/journal.pcbi.1001093)

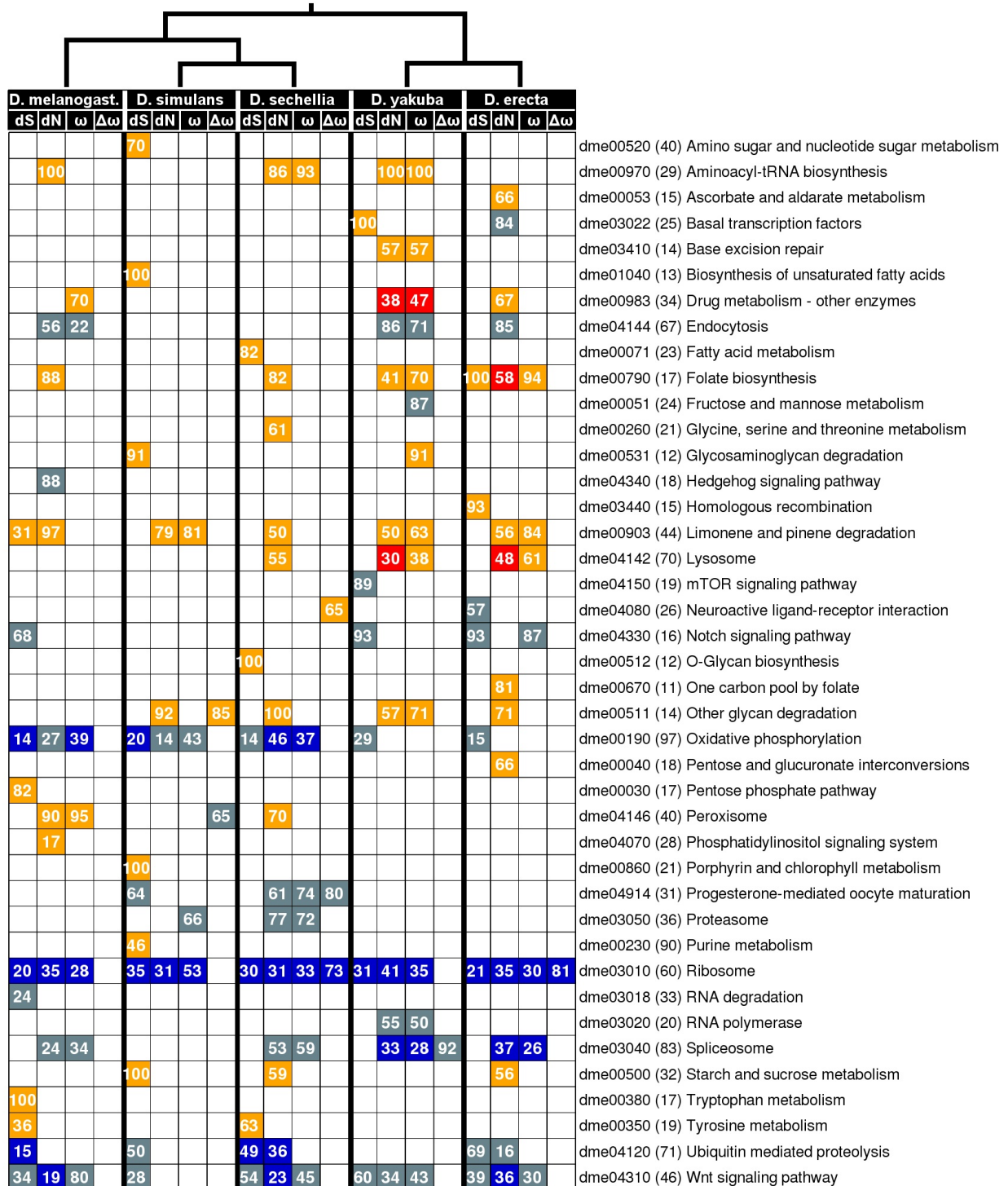

Supplement: Figure S4 — Complete list of significant results of GSSA for KEGG pathways in Drosophila species. GSSA (5% FDR) results (43 KEGG pathways) for dS, dN, ω & Δω in Drosophila species. (0.87 MB PDF) [file pcbi.1001093.s004.pdf]

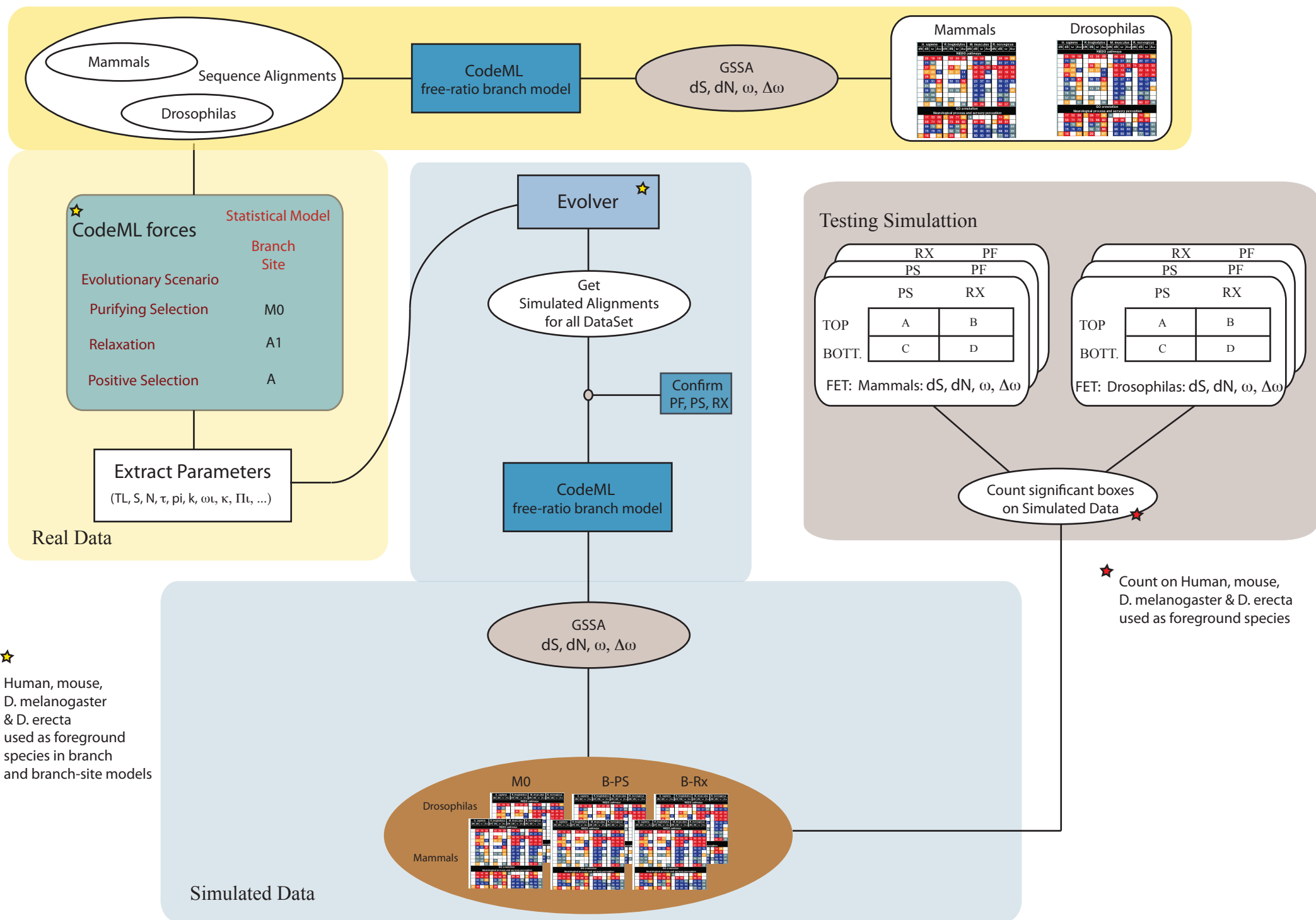

Supplement: Figure S6 — Evolutionary and statistical simulation of GSSA. The pipeline shows the steps taken along three different spaces of analysis, the real data, the simulated data and the testing block. See Supplementary Results for a complete explanation of methods and results. (1.42 MB PDF) [file pcbi.1001093.s006.pdf]
