# Supplementary material for: Natural Selection on Functional Modules, a Genome-Wide Analysis
Source: PLoS Comput Biol. 2011 Mar 3;7(3):e1001093. doi: 10.1371/journal.pcbi.1001093 (PMC3048381; doi:10.1371/journal.pcbi.1001093)
Supplement: Figure S5 — Randomisation experiment. (A) The pipeline shows the steps followed to tests possible biases attributed to the size of the functional category, the magnitude of change in evolutionary rate and the proportion of genes experiencing a rate change in the GSSA. The proportion of false positive results never reached 5% (FDR) in mammals (B) and Drosophila (C). (1.34 MB PDF) [file pcbi.1001093.s005.pdf]

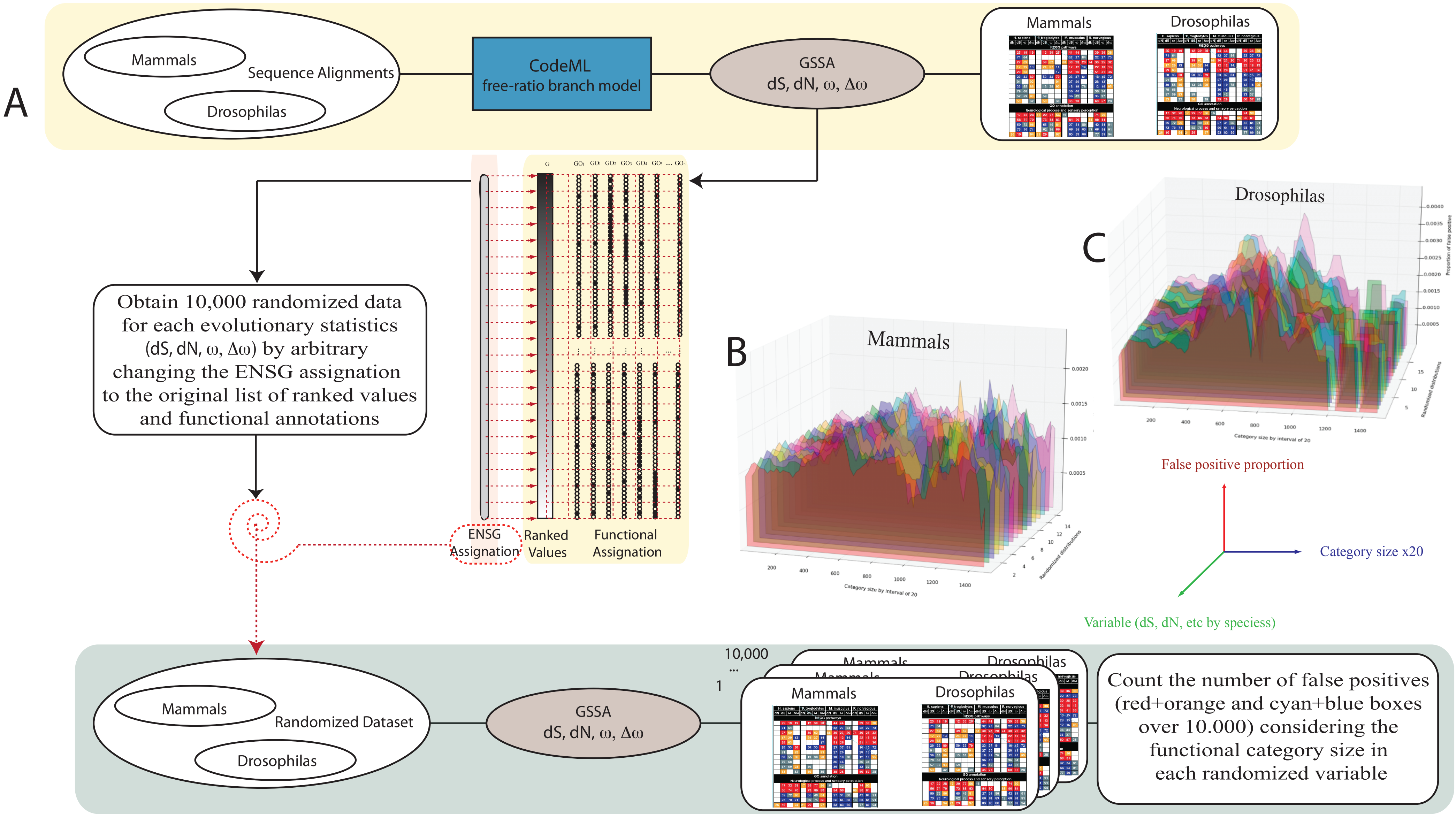
A
Mammals
Sequence Alignments
Drosophilas
CodeML
free-ratio branch model
GSSA
dS, dN, ω, Δω
Mammals
Drosophilas
Obtain 10,000 randomized data for each evolutionary statistics (dS, dN, ω, Δω) by arbitrary changing the ENSG assignation to the original list of ranked values and functional annotations
ENSG Assignation
Ranked Values
Functional Assignation
B
Mammals
Category size by interval of 20
Randomized distributions
C
Drosophilas
Proportion of false positive
False positive proportion
Category size x20
Variable (dS, dN, etc by speciess)
Mammals
Randomized Dataset
Drosophilas
GSSA
dS, dN, ω, Δω
10,000
...
1
Mammals
Drosophilas
Count the number of false positives (red+orange and cyan+blue boxes over 10.000) considering the functional category size in each randomized variable

B

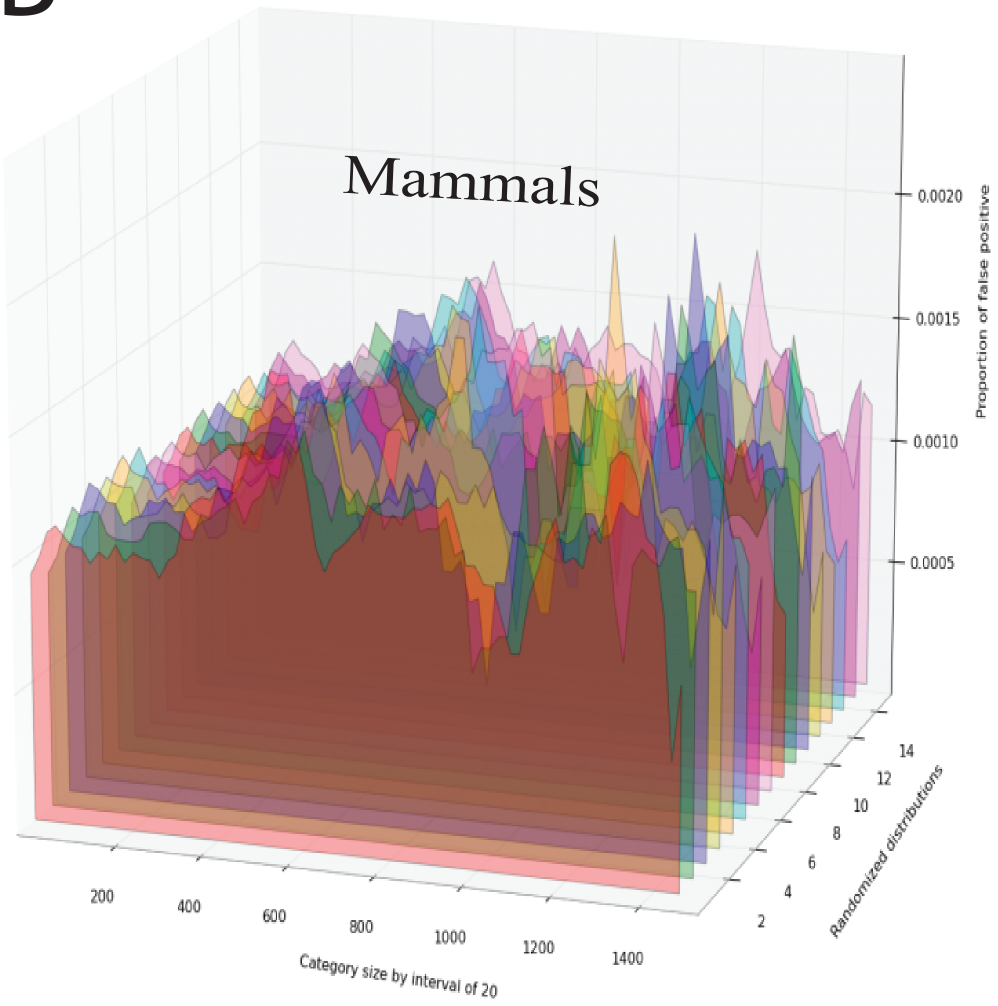
Mammals
0.0020
0.0015
0.0010
0.0005
Proportion of false positive
200
400
600
800
1000
1200
1400
Category size by interval of 20
2
4
6
8
10
12
14
Randomized distributions

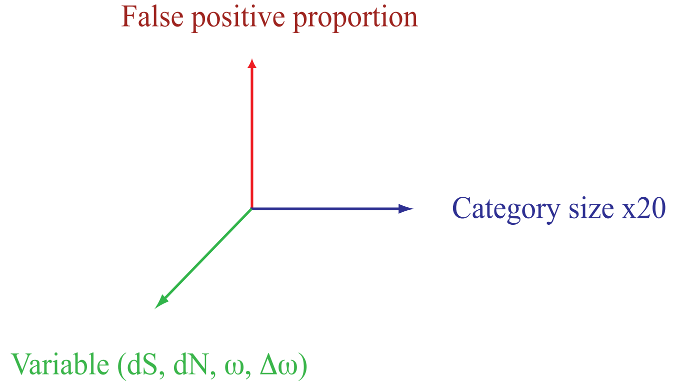
False positive proportion
Category size x20
Variable (dS, dN, ω, Δω)

C

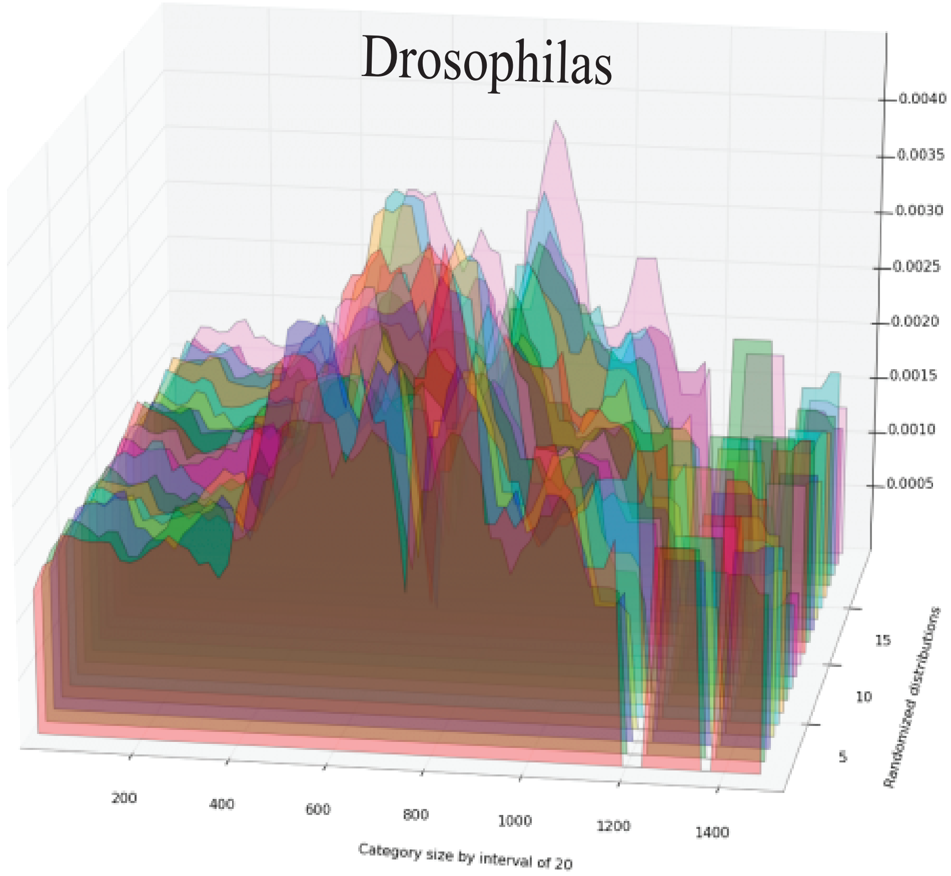
Drosophilas
0.0040
0.0035
0.0030
0.0025
0.0020
0.0015
0.0010
0.0005
Proportion of false positive
200
400
600
800
1000
1200
1400
Category size by interval of 20
5
10
15
Randomized distributions
