# Supplementary material for: Natural Selection on Functional Modules, a Genome-Wide Analysis
Source: PLoS Comput Biol. 2011 Mar 3;7(3):e1001093. doi: 10.1371/journal.pcbi.1001093 (PMC3048381; doi:10.1371/journal.pcbi.1001093)
Supplement: Table S3 — The complete functional enrichment results using gene-by-gene and gene-set approaches. The table depicts all the biological functions enriched by PSGs as cited in references 1 to 7, and the corresponding significant result observed after GSSA of ω values. References 1 to 7 correspond to cites 6, 7, CSAC, 4, 5, 9 and 8 in the manuscript, respectively. Abbreviations: SHω: statistically significant high ω values; SLω: statistically significant low ω values; H: human; C: chimpanzee; Pr: primates; M: mouse; R: rat; Ro: rodents; Dmel: D. melanogaster; Dsim: D. simulans; Dsec: D sechelia; Dyak: D. yakuba; Dere: D. erecta; Ds: Drosophila species. *: p<0.05; ** p<0.001. CSAC: Chimpanzee Sequencing and Analysis Consortium, Nature. 2005 vol. 437 (7055) pp. 69–87. (0.09 MB PDF) [file pcbi.1001093.s009.pdf]

Biological process	Functional category enriched by PSGs (Reference #)							GSSA results	
	1	2	3	4	5	6	7	SHω	SLω
Olfaction / Sensory perception of smell	H	Pr**				Pr**		H**, C**, M**, R** Dmel*, Dsec*, Dere**, Dyak**	
Chemosensory perception	H	Pr**						H**, C**, M**, R** Dmel**, Dsec**, Dere**, Dyak**	
G-protein-mediated signaling	H				H	Pr**		H**, C**, R*	
DNA/nucleic acid metabolism				C			Ds	C*, M**, R**, Dmel**, Dyak**, Dere*	
Amino acid metabolism	H, C						Ds	M**, R**	
Proteolysis							Ds	M**, R**, Dmel**, Dsim*, Dsec*, Dyak**, Dere**	
Fatty acid/Lipid metabolism					H		Ds	M**, R**	
Carbohydrate metabolism							Ds	Dsec*, Dyak*, Dere*	
Adult reproduction and gametogenesis							Ds	Dsec*	
Spermatogenesis and motility		Pr*	Pr					H*, M*, Dmel*	
Immune response		Pr**		H, C		Ro**		C*, M**, R**, Dyak*, Dere*	
Inflammatory response						Ro**		H*, C*, M**, R**	
Defense response						Ro**		H*, C*, M**, R**, Dyak**, Dere*	
Response to wounding						Ro**		H*, M**, R**	
Hummoral immune response mediated by circulating immunoglobulin						Ro**		M**, R**	
T-cell-mediated immunity		Pr**						M*	
Natural killer-cell-mediated immunity		Pr*						R*	
B-cell- and antibody-mediated immunity		Pr*						M**, R**	
Response to pest, pathogen, or parasite				H				C*, M**, R**, Dyak*, Dere*	
Stress response					C	Ro**		M**, R**	
Response to external stimulus						Ro**		M**, R*	
Sensory Perception	H	Pr**		H		Pr**		H**, C**, M*, Dmel**, Dsec**, Dyak**. Dere**	R*
Cell surface receptor-mediated signal transduction	H					Pr**		C*	Dmel*, Dyak*, Dere*
Cell adhesion	H							R*	H**, C**, Dmel**, Dere*
Amino acid transport			Pr					M*	R*
Protein amino acid glycosylation			Pr					M*	H*
Amino acid transport	C							M*	C*
Hearing / Perception of sound	H		Pr						M*, R*
Neurological process						Pr**			M**, R**, Dyak*, Dere*
Synaptic transmission			Pr						H**, M**, R**, Dmel**, Dsec**, Dere**, Dyak**
Signal transduction/intracellular signaling cascade	H, C		Pr				Ds		H**, C**, M**, R**, Dmel**, Dsec*, Dyak**, Dere**
Ion transport	H				H		Ds		H*, M**, R**, Dmel*, Dsec*, Dere*
Potassium ion transport			Pr						H*, C*, M**, R**
Inorganic anion transport			Pr						M*, R*

Intracellular protein traffic	H								H**, C**, M**, R**,<br>Dmel*, Dsec**, Dyak**, Dere*
Transport							Ds		Dmel**, Dsec**, Dere**, Dyak**
Protein transport				H			Ds		H*, C**, M**, R**,<br>Dmel**, Dsim*, Dsec**, Dere**, Dyak**
Metabolism of cyclic nucleotides	H								M*, R*
Protein metabolism & modification				H, C	C		Ds		H**, C**, M**, R**, Dere*, Dyak*
Phosphate metabolism/phosphorylation				H, C			Ds		H*, C*, M**, R**,<br>Dmel*, Dsec**, Dyak**, Dere*
Purine metabolism	C								M*, R*, Dsec**
Carbohydrate biosynthesis			Pr						M**, R*
Cation transport	H								H*, M**, R**
Nervous system development							Ds		H*, M**, R**,<br>Dmel**, Dsec*, Dyak**, Dere**
Skeletal development	C								M**, R**
Organ development							Ds		H*, M**, R**,<br>Dmel**, Dsec*, Dyak**, Dere**
Post-embryonic development							Ds		M*, Dmel*, Dyak**, Dere*
Embryonic development							Ds		H**, C*, M**, R**, Dyak*, Dere*
Ectoderm development					H				C*, M*, R*, Dmel*, Dyak*, Dere*
Cell proliferation and differentiation	C						Ds		H**, C*, M**, R**,<br>Dmel**, Dsec*, Dyak**, Dere**
Cell cycle							Ds		H*, M*, R*,<br>Dmel**, Dsec**, Dyak**, Dere**
Cell structurre/morphogenesis	C						Ds		H**, C*, M**, R**,<br>Dmel**, Dsec*, Dyak**, Dere**
Cell structure and motility	C								H*, M**, R**, Dsec*
Inhibition of apoptosis		Pr*							H*, Dyak*
Cell-cell signalling							Ds		H**, C*, M**, R**,<br>Dmel**, Dsec**, Dere**, Dyak**
Regulation of nucleobase				H, C					H**, C**, M**, R**, Dere*
Translation							Ds		M*, R*,<br>Dmel**, Dsim*, Dsec**, Dyak**, Dere**
Transcription				H, C	C		Ds		H**, C**, M**, R**, Dere*
Protein catabolism				H, C	C				H**, C**, M**, R**
Lipid transport			Pr						
Phosphate transport					H				
Oncogenesis	C								
Interferon-mediated immunity		Pr*							
