# Supplementary material for: Natural Selection on Functional Modules, a Genome-Wide Analysis
Source: PLoS Comput Biol. 2011 Mar 3;7(3):e1001093. doi: 10.1371/journal.pcbi.1001093 (PMC3048381; doi:10.1371/journal.pcbi.1001093)

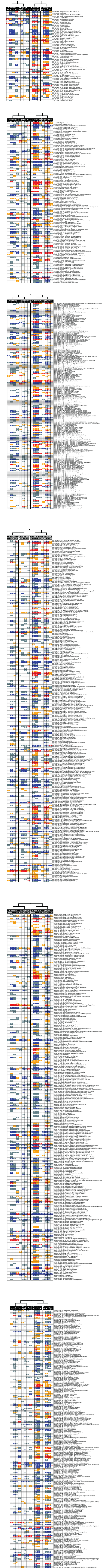

Supplement: Figure S1 — Complete list of significant results of GSSA for GO terms in mammals. The figures cover from the most general to the most specific biological GO functions. GSSA (5% FDR) results for dS, dN, dN/dS & Δω using 731 GO terms. (6.47 MB PDF) [file pcbi.1001093.s001.pdf]
