# Supplementary material for: Natural Selection on Functional Modules, a Genome-Wide Analysis
Source: PLoS Comput Biol. 2011 Mar 3;7(3):e1001093. doi: 10.1371/journal.pcbi.1001093 (PMC3048381; doi:10.1371/journal.pcbi.1001093)
Supplement: Text S1 — Supplementary Text S1. (0.11 MB PDF) [file pcbi.1001093.s010.pdf]

Supplementary Text S1

Natural selection on functional modules, a genome-wide analysis

by François Serra, Leonardo Arbiza, Joaquín Dopazo and Hernán Dopazo

Evolutionary Simulation.

Methods

The pipeline described in Figure S6 shows three different areas: the real data, the simulated data and the testing block.

Real Data: the dark yellow area describes the steps used to reach to results described in the manuscript. The light yellow area describes the use of the CodeML program from PAML package (reference 15 in the ms) to extract -from the original set of sequences- the evolutionary parameters to simulate new sequences under purifying selection (PF), positive selection (PS) and relaxation of the selective constraints (RX) using branch-site models (see *model description* below). Human, mouse, *D. erecta* and *D. melanogaster* were used as foreground species in the corresponding models.

Simulated Data: Evolver (PAML program) simulates sequences using parameters (codon frequencies and branch lengths) from the empirical data. We checked the desired characteristics of positive selection (PS) and relaxation of selective constraints (RX) on the set of the simulated sequences (Table A). Evolutionary variables (dS, dN, ω and $\Delta\omega$) were estimated from simulated sequences by means of a free-ratio branch model (CodeML). The complete pipeline of the Gene-Set Selection Analysis (GSSA described in the ms) was applied in the simulated data.

Testing simulations: The odd-ratio of the values observed on the contingency table of each significant functional term after GSSA was computed[1]. Values higher and lower than one contribute to the total number of functional modules with significant high and low ω values. To test the statistical contribution of these functional modules to these extremes on the simulated regimes (PS, RX and PF) the log odd-ratios were compared using a t-test in the R statistical package.

Results

Our results showed that in spite of the alternative evolutionary scenarios no significant differences were observed between log odd-ratios distribution ($p<0.05$). This result is exactly what we expected. The average effect of PF, and RX-PS is the proportional decrease and increase of the mean value of ω on sequences, respectively. This change has minor effects (if any) in the relative position of genes in the ranked list of genes of a genome. Accordingly, since no net differences were produced after ranking genes, no significant differences are expected after the t-test (PS-RX: $p= 0.99$, PS-PF: $p= 0.45$, and RX-PF: $p= 0.46$). The fact that basically the same number of significant results was observed in each evolutionary scenario

[1] Using the nomenclature of Figure 2 this is (GO-A/Rest-A)/(GO-B/Rest-B)

confirmed this prediction (Table B). We conclude that neither of the selective regimes simulated produce significant differences or biases in the GSSA of ω values.

Table A
Number of PSG and relaxed genes (RXG) in each of the simulated evolutionary scenarios

	PS		RX		PF	
	# PSG	# RXG	# PSG	# RXG	# PSG	# RXG
Homo sapiens	658	1640	11	1939	0	1
Mus musculus	1500	954	14	1565	1	0
D. melanogaster	736	630	25	1104	0	0
D. erecta	778	1292	26	1713	2	1

Table B. Proportion of significant functional categories that are still significant (identical signs of odd-ratios) under a different evolutionary scenario.

	PS	RX	PF
PS	---	92.50%	98.50%
RX	91.10%	---	99.00%
PF	88.90%	90.60%	---

Models Description

Branch-site models (M0, A, A1).

To simulate purifying selection in the M0 model we used a single site class (0) on all the branches of the tree. Branch-site model A assumes ω ratios according to the observed in the Table (Zhang, et al 2005, reference 31 in the ms) to simulate PS on the sequences. Branch-site model A1 used to simulate sequences under relaxation of selective constraints assumes identical values in the background but ω2a and ω2b are constrained to 1 in the foreground.

Site class	Proportion	Background ω	Foreground ω
0	p_0	$0 < \omega_0 < 1$	$0 < \omega_0 < 1$
1	p_1	$\omega_1 = 1$	$\omega_1 = 1$
2a	$(1 - p_0 - p_1)p_0/(p_0 + p_1)$	$0 < \omega_0 < 1$	$\omega_2 > 1$
2b	$(1 - p_0 - p_1)p_1/(p_0 + p_1)$	$\omega_1 = 1$	$\omega_2 > 1$
